# Transcryptomic Analysis of Human Brain -Microvascular Endothelial Cell Driven Changes in -Vascular Pericytes

**DOI:** 10.3390/cells10071784

**Published:** 2021-07-14

**Authors:** Lisa Kurmann, Michal Okoniewski, Raghvendra K. Dubey

**Affiliations:** 1 Department of Reproductive Endocrinology, University Hospital Zurich, 8952 Schlieren, Switzerland; lisa.kurmann@usz.ch; 2 ID Scientific IT Services, ETH Zurich, 8092 Zurich, Switzerland; michal.okoniewski@id.ethz.ch; 3 Department of Pharmacology & Chemical Biology, University of Pittsburgh, Pittsburgh, PA 15219, USA

**Keywords:** hCMEC/D3, blood–brain barrier, transcriptome, microarray, inflammation, co-culture

## Abstract

Many pathological conditions of the brain are associated with structural abnormalities within the neurovascular system and linked to pericyte (PC) loss and/or dysfunction. Since crosstalk between endothelial cells (ECs) and PCs greatly impacts the function of the blood–brain barrier (BBB), effects of PCs on endothelial integrity and function have been investigated extensively. However, the impact of ECs on the function and activity of PCs remains largely unknown. Hence, using co-cultures of human brain vascular PCs with human cerebral microvascular ECs on opposite sides of porous Transwell inserts which facilitates direct EC–PC contact and improves EC barrier function, we analyzed EC-driven transcriptomic changes in PCs using microarrays and changes in cytokines/chemokines using proteome arrays. Gene expression analysis (GEA) in PCs co-cultured with ECs versus PCs cultured alone showed significant upregulation of 1′334 genes and downregulation of 964 genes. GEA in co-cultured PCs revealed increased expression of five prominent PC markers as well as soluble factors, such as transforming growth factor beta, fibroblast growth factor, angiopoietin 1, brain-derived neurotrophic factor, all of which are involved in EC–PC crosstalk and BBB induction. Pathway enrichment analysis of modulated genes showed a strong impact on many inflammatory and extracellular matrix (ECM) pathways including interferon and interleukin signaling, TGF-β and interleukin-1 regulation of ECM, as well as on the mRNA processing pathway. Interestingly, while co-culture induced the mRNA expression of many chemokines and cytokines, including several CCL- and CXC-motif ligands and interleukins, we observed a decreased expression of the same inflammatory mediators on the protein level. Importantly, in PCs, ECs significantly induced interferon associated proteins (IFIT1, IFI44L, IF127, IFIT3, IFI6, IFI44) with anti-viral actions; downregulated prostaglandin E receptor 2 (prevent COX-2 mediated BBB damage); upregulated fibulin-3 and connective tissue growth factor essential for BBB integrity; and multiple ECMs (collagens and integrins) that inhibit cell migration. Our findings suggest that via direct contact, ECs prime PCs to induce molecules to promote BBB integrity and cell survival during infection and inflammatory insult. Taken together, we provide first evidence that interaction with ECs though porous membranes induces major changes in the transcriptomic and proteomic profile of PCs. ECs influence genes involved in diverse aspects of PC function including PC maturation, cell survival, anti-viral defense, blood flow regulation, immuno-modulation and ECM deposition.

## 1. Introduction

Embryonic development as well as vascular homeostasis in adulthood strongly depends on the intercellular communication between different cell types of the vascular system. In the brain, the neurovascular unit (NVU) comprises different cell types, all of which are indispensable for the integrity of the blood–brain barrier (BBB) and proper neural function [1]. Next to the innermost lining of the blood vessels, which is formed by a single layer of endothelial cells (ECs), astrocytes and pericytes (PCs) are essential players of the NVU [2,3]. The importance of PCs with regard to CNS homeostasis has repeatedly been shown by animal knock-out as well as in vitro studies [3] and, importantly, PC loss is correlated with many cardiovascular and CNS disorders [4,5]. Abnormal PC recruitment is also observed in tumor tissue and the degree of PC coverage has been related to clinical outcome and metastasis formation [6].

PCs are vascular mural cells that are embedded in the same basement membrane as ECs [3]. Interaction of the two cell types by paracrine signaling involves soluble factors such as transforming growth factor beta (TGF-β), vascular endothelial growth factor (VEGF), sphingosine-1-phosphate (S1P), platelet-derived growth factor-beta (PDGF-B) and angiopoietin 1 and 2 (Ang1/Ang2), all of which constitute an important part in regulating endothelial integrity [1,4,7]. Furthermore, an essential role in the communication between ECs and PCs is dedicated to the direct contacts between the two cell types, so-called peg–socket interactions harboring gap junctions and adhesion plaques [3,8]. These direct contacts allow the exchange of small metabolites, ions and second messengers between two neighboring cells. One PC is thought to contact several ECs, thereby being responsible for the important task of integrating EC responses [3]. Additionally, both cell types make fundamental contributions to the composition of the basement membrane and secrete extracellular matrix (ECM) proteins such as laminin, collagen and fibronectin among many others [9]. Basement membrane composition is highly versatile and undergoes dynamic changes along with different developmental stages and according to the cellular environment [10].

Next to their important and well investigated function in endothelial barrier formation, PCs undertake other fundamental tasks including regulation and maintenance of vascular tone, tissue repair, phagocytosis and immunomodulatory actions [11]. In this regard, it has been demonstrated that cytokine-stimulated PCs show increased phagocytic activities [12] in order to clear cellular debris and invading pathogens [13]. Additionally, PCs are capable of reducing plaque deposition in Alzheimer’s disease by clearing amyloid beta peptides [14]. Activated PCs exhibit an upregulated secretion of a wide range of chemokines and cytokines, thereby evoking responses in a variety of cell types of the innate and adaptive immune system and further impact inflammation-induced angiogenesis [15,16]. This wide facet of PC’s inflammatory secretome highlights their importance in neuroinflammation and immunomodulation. While neuroinflammation is an important process in the defense against pathogens and toxins, uncontrolled and chronic inflammatory processes increase endothelial dysfunction and will ultimately lead to neuronal damage [17].

Most research has focused on assessing the effects of mural and glial cells on endothelial integrity and barrier function, including a recent publication from our lab [18]. However, much less is known about the impact of ECs on mural cell function, including PCs. With increasing importance of the role of PCs in the neurovascular unit, the aim of this study was to elucidate the impact of human brain microvascular ECs on human brain vascular PCs. To accomplish our goals, transcriptomic analysis using microarray was performed on PCs cultured alone or in co-culture with ECs on opposite sides of permeable Transwell inserts, which allows ECs and PCs to have direct cell–cell contact and permits the exchange of paracrine factors through the pores of the membrane, as shown by Kang et al. [16]. In the present study, we specifically investigated EC driven changes in PC gene expression using the EC–PC co-culture model that facilitates EC-barrier function, as we previously demonstrated [18]. Additionally, we performed cytokine proteome arrays, to elucidate whether the observed transcriptional changes in the inflammatory profile of PCs are also reflected on the protein level.

## 2. Materials and Methods

### 2.1. Cell Culture

hBVPs: Human Brain Vascular Pericytes (HBVPs, ScienCell, CA, USA) between 4th and 10th passage were cultured in Poly-L-Lysine- (PLL-) coated flasks (2 µg/cm^2^) under standard tissue culture conditions (37 °C, 5% CO_2_) in growing media consisting of DMEM/F12 supplemented with antibiotic-antimycotic (AA; 100 μg/mL streptomycin, 100 μg/mL penicillin and 0.025 μg/mL amphotericin B), Glutamax (1×) and 10% FBS. Media was changed every two or three days until sub-confluency.

hCMEC/D3: The human Cerebral Microvascular Endothelial Cell line (hCMEC/D3) [19] was kindly provided by Dr. Pierre-Olivier Couraud (Institute COCHIN, Paris, France). Cells between 34th and 39th passage were cultured on rat-tail-collagen-coated (250 μg/mL in 80% EtOH) flasks under standard tissue culture conditions (37 °C, 5% CO_2_) in complete growing media (EC basal media (EndoGRO Basal Medium supplemented with 0.2% EndoGRO-LS Supplement, 5 ng/mL rh EGF, 4 mM L-Glutamine, 0.75 U/mL Heparin Sulfate, 50 μg/mL Ascorbic Acid, 1 ng/mL bFGF, antibiotic-antimycotic (100 μg/mL streptomycin, 100 μg/mL penicillin and 0.025 μg/mL amphotericin B))) supplemented with 5% FBS. Media was changed every two or three days and cells were passaged after confluency was reached.

### 2.2. Immuno-Fluorescence

PCs were grown on PLL-coated glass slides. Cells were fixed by adding paraformaldehyde (4%) to the media (1:1) for 2 min at RT without shaking, before media was removed and replaced by PFA (4%) for an additional 15 min. Subsequently, cells were washed 3 × 5 min with PBS and permeabilized with 0.1% Triton-X in PBS for 5 min at RT if necessary. Cells were washed 3 × 10 min with PBS. Normal goat serum (NGS, 10% in PBS) was used for blocking for 90 min at RT while shaking slightly. Primary antibody diluted in 2% NGS in PBS/T (0.2% Tween 20) was added for overnight at 4 °C. Subsequent washing (3 × 10 min in PBS/T) was followed by the addition of secondary antibody diluted 1:500 in 5% NGS-PBS/T for 90 min at RT. Cells were then washed 1× with PBS/T and 2× with PBS and stained with DAPI (100 ng/mL) for 2 min at RT. Cells were washed again 2× with PBS before images were taken in freshly stained cells without any mounting media by using an Olympus IX81 microscope (Olympus, Volketswil, CH, Switzerland).

### 2.3. Microarray Analysis

For the microarray samples, ECs were seeded alone or with PCs on the opposite side of permeable PET membrane inserts of 0.4 µm pore size and six-well format (Corning Incorporated, Corning, NY, USA, Costar 3450). After 7 days in culture (2% steroid-free FCS (charcoal-stripped) in presence of hydrocortisone), cells were trypsinized, centrifuged and lysed in 300 μL RNA lysis buffer (Zymo Research, Irvine, CA, USA). Samples were frozen at −80 °C until further processing. Total RNA was then extracted by using the Quick-RNA MiniPrep Kit (ZymoResearch, CA, USA, R1055) according to the manufacture’s protocol. RNA integrity was checked by calculating the ratio of absorbance at 260 nm and 280 nm/230 nm respectively. The samples were frozen at −80 °C. Microarray analysis using Affymetrix Clariom S Assay, human (Applied Biosystems by Thermo Fisher Scientific Inc, Waltham, MA, USA, 902927) was performed as previously described [20]. For transcriptome analysis, fragmented biotin-labeled ds cDNA was hybridized to Clariom™ S arrays (Clariom™ S arrays, human). After staining, arrays were scanned with Affymetrix Gene-Chip Scanner-3000-7G (Applied Biosystems by Thermo Fisher Scientific Inc, Waltham, MA, USA) while quality control analysis was performed using GeneChip Command Console Software (GCC) v5.0. Transcriptome analysis was done at the transcriptomics core facility at the Center for Molecular Medicine Cologne (CMMC). Differentially regulated genes were determined with the Transcriptome Analysis Console (TAC, Applied Biosystems by Thermo Fisher Scientific Inc, Waltham, MA, USA) after uploading the CEL files, based on a foldchange cut-off of +/− 1.5 (Log2 FC +/− 0.59) and FDR *p*-value of 0.05. Pathway analysis was performed using NCATS BioPlanet on the Enrichr website, which includes more than 1600 human pathways from publicly available sources [21]. Additionally, enrichment analysis with GO biological processes and KEGG pathways have been performed also on the Enrichr website. The microarray data are deposited in the public Gene Expression Omnibus (GEO) database under the accession no. GSE168514 (Available online: www.ncbi.nlm.nih.gov/geo/query/acc.cgi?acc=GSE168514) (accessed on 12 May 2021).

### 2.4. Quantitative RT-PCR

Sample preparation and RNA isolation for the verification of microarray results by means of quantitative real-time polymerase chain reaction (qRT-PCR) was performed exactly as described in the microarray section above. RNA integrity was checked by calculating the ratio of absorbance at 260 nm and 280 nm/230 nm. The samples were frozen at −80 °C until further use. cDNA was synthesized from 0.5 µg total RNA using the RT^2^ First Strand Kit from Qiagen according to manufacturer’s instruction. Relative gene expression levels were determined by using Custom RT^2^ Profiler PCR Arrays in a 96-well-plate format according to the manufacturer’s instruction on a CFX96 real-time PCR detection system (BioRad, CH). Incubation at 95 °C for 10 min was followed by 40 cycles of 95 °C for 15 s and 60 °C for 1 min. For data normalization, GAPDH and PDGFRB were used as internal controls. The catalogue numbers of the used genes/primers can be found in the supplementary data. The relative gene expression was calculated using the 2^−^^ΔΔCt^ method.

### 2.5. Cytokine Proteome Array

For analysis of cytokine expression in co- and mono-cultured PCs the Proteome Profiler Human XL Cytokine Array Kit (R&D Systems, MN, USA, ARY022B) was used. Cells were grown exactly as described in the microarray section above. After trypsinization and centrifugation, the pellet was lysed in lysis buffer 17 (R&D Systems) supplemented with aprotinin, leupeptin and pepstatin (10 µg/mL each) and after processing according to the user’s manual, they were frozen at −80 °C until further use. Incubation of the ready-to-use membranes was performed overnight with equal amounts of samples (120 µg in 1.5 mL), as was determined by BCA analysis. The following detection of the proteins was performed exactly as described in the user’s manual and for the exposure of the membranes Hyperfilm ECL (Amersham, CH, Switzerland) were used in a CAWOMAT 2000 IR film developer (Wiroma AG, Niederscherli, CH, Switzerland). Experiments were performed two times with independently prepared samples. The signal density of each spot was determined with the imageJ software after background subtraction and values represented in the graphs denote mean values of each protein from the two experiments.

### 2.6. Western Blot Analysis

For Western blot analysis, cells were grown exactly as described in the microarray section above. After trypsinization and centrifugation, the pellet was lysed in lysis buffer (containing 20 mM Tris pH 7.5, 1% Triton X- 100, 150 mM NaCl, 1 mM EGTA, 1 mM EDTA, 2.5 mM sodium phosphate, 1 mM β-glycerophosphate, 1 mM sodium vanadate, 0.5 PMSF and 0.2% SDS). Concentration of each sample was determined with the Pierce bicinchoninic acid (BCA) assay kit according to the manufacturer’s protocol. Equivalent amounts (10 μg) of protein from whole-cell lysates were separated on 10% SDS-polyacrylamide gels. After transfer to a nitrocellulose membrane by the method of wet electroblotting, the membrane was blocked with 5% milk at RT for 1 h. Incubation with the primary antibody was performed overnight at 4 °C. After washing, the membrane was incubated with the secondary antibody for 1 h at RT and washed again. For detection of proteins with IR Dyes, the Odyssey LI-COR system (LI-COR, Lincoln, NE, USA) was used. For peroxidase-conjugated secondary antibodies, chemiluminescent substrates (Pierce Biotechnology Inc. by Thermo Fisher Scientific Inc, Waltham, MA, USA,) were added according to manufacturer’s instruction. Peroxidase activity was detected by exposing the membranes to XOMAT LS films, which were developed with the CAWOMAT 2000 IR film developer (WIROMA AG, Niederscherli, CH, Switzerland).

### 2.7. Statistical Analysis

Microarray data analysis was performed with the Transcriptome Analysis Console (TAC, Applied Biosystems), which uses generalized linear models (Limma-based, eBayes). The generation of probe set intensity values was performed by the signal space transformation (SST)-RMA normalization method and background correction. Differentially regulated genes (DRGs) were determined based on a foldchange cut-off of +/− 1.5 (Log2 FC +/− 0.59) and FDR *p* value of 0.05.

## 3. Results

### 3.1. Differentially Regulated Genes

**Figure 1:**
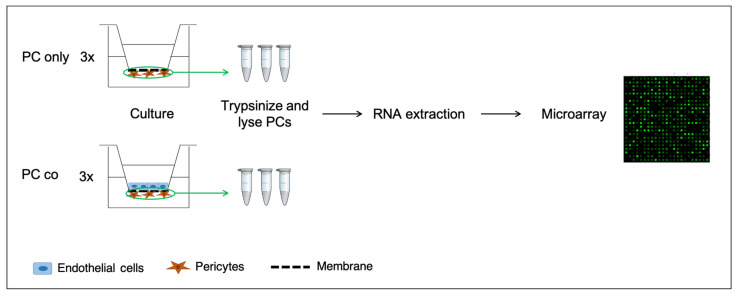
Schematic illustration of the experimental setup. Pericytes (PCs) are grown on the basolateral side of 24-well plate inserts alone (PC only) or in co-culture with ECs on the apical side with direct contact. After 7 days in culture, cells were trypsinized and lysed for total RNA extraction, before microarray analysis was performed.

**Figure 2:**
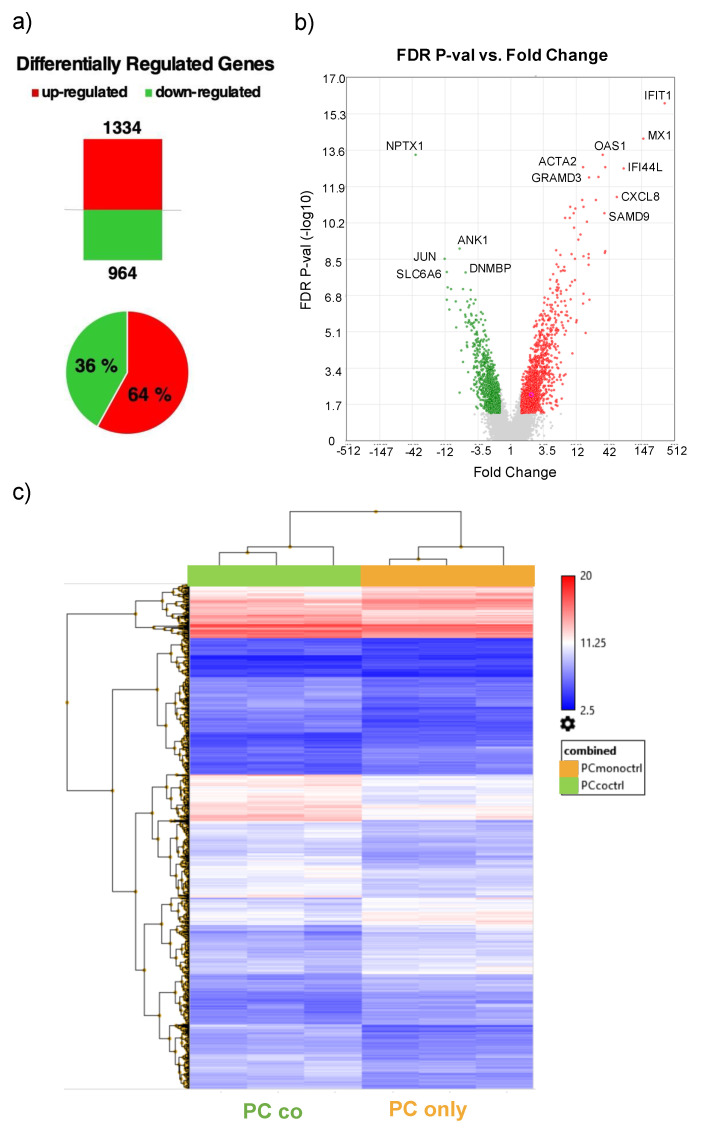
Differentially regulated genes (DRGs). Number of differentially regulated genes (DRGs) in PCs co-cultured with ECs vs. PCs cultured alone and pie chart representation denoting up- and downregulated genes in % of total number of DRGs. Upregulated genes are depicted in red and downregulated genes in green (**a**). Volcano plot showing FDR *p*-Value (−log10) on the *y*-axis vs. fold change of DRGs on the *x*-axis. Up- and downregulated genes are depicted in red and green, respectively (**b**). Heatmap representation of DRGs between co- and mono-cultured PCs (**c**). Comparison of gene expression data of co-cultures vs. mono-cultures of PCs in triplicates was performed by using Transcriptome Analysis Console (TAC, Applied Biosystems by Thermo Fisher Scientific Inc, Waltham, MA, USA)). For the analysis, a fold change (FC) cut-off of 1.5 (log2FC +/− 0.59) and FDR *p*-Value of 0.05 was applied.

**Figure 3:**
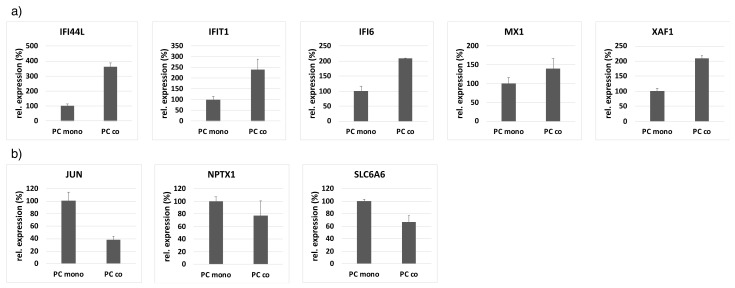
Confirmation of the most highly regulated genes (upregulated (**a**): IFI44L, IFIT1, IFI6, MX1, XAF1; downregulated (**b**): JUN, NPTX1, SLC6A6) in the microarray by qRT-PCR by using custom-designed RT2 PCR arrays from Qiagen. For data normalization, GAPDH and PDGFRB were used as internal controls, the expression of which was shown to be not regulated in the microarray. The experiment was performed one time in triplicates and data represent mean +/− standard deviation of the mean.

By comparing the gene expression profile of pericytes (PCs) cultured alone or in co-culture with endothelial cells (ECs) by microarray analysis (Figure 1), a total of 2’239 differentially regulated genes (DRGs) was detected. Of these DRGs, 1’334 were up- and 964 were downregulated in co-cultured cells (Figure 2a). Principle component analysis shows a clear separation of the expression profiles of mono- and co-cultured PCs (Supplementary Figure S1). A list of the top ten genes up- and downregulated in co-cultured PCs is provided in Table 1 and Table 2. The most highly regulated genes have been confirmed by quantitative RT-PCR. The highly upregulated genes IFI44L, IFIT1, MX1, XAF1 and IFI6 from the microarray also showed increased expression in the PCR, while the downregulated expression profile of NPTX1, JUN and SLC6A6 was also confirmed (Figure 3, Supplementary Table S1).

Pathway enrichment analysis on DRGs using NCATS Bioplanet revealed a high impact of co-culture on pathways involved in extracellular matrix (ECM) regulation induced by transforming growth factor-beta (TGF-β) and interleukin-1 as well as several inflammatory signaling related pathways. Additionally, epidermal growth factor receptor 1 (EGFR1) and brain-derived neurotrophic factor (BDNF)-signaling pathways are regulated, as well as pathways of lipid metabolism regulation and mRNA processing (Table 3). Comparing pathway enrichment analysis from Bioplanet with GO Biological Processes and KEGG pathways (Supplementary Tables S2 and S3) we found an overlap of the following pathways in at least two of the three enrichment analysis: interferon signaling and viral pathways, mRNA processing, regulation of gene expression, autophagy, transforming growth factor (TGF) -beta regulation, vascular smooth muscle cell contraction and ECM/proteoglycans.

### 3.2. Assessment of Pericyte Marker Expression

**Figure 4:**
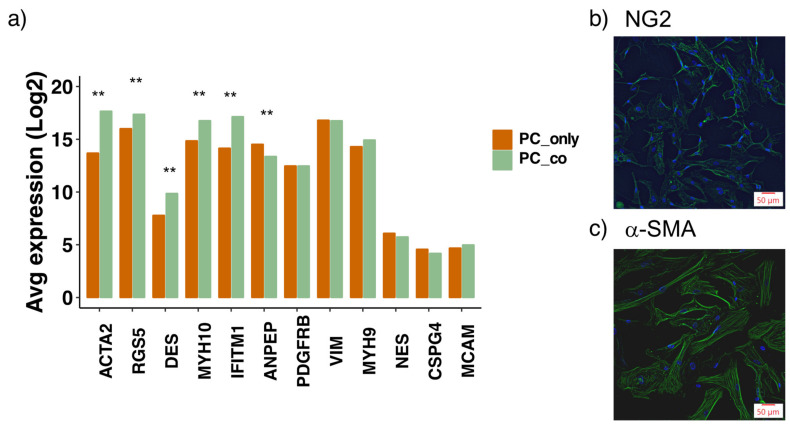
Transcriptional expression of commonly used pericyte (PC) marker genes in pericytes and their changes upon co-culture with endothelial cells (ECs). Log2 expression levels of PC marker genes are depicted from PCs cultured alone (PC_only) and together with ECs (PC_co) (**a**). Data represent mean as derived from microarray data analysis that was performed in triplicates for each condition. Data analysis was performed by using Transcriptome Analysis Console (TAC, Applied Biosystems). ** FDR *p*-Value < 0.01, compared to PC_only. Immunofluorescent images of PCs showing expression of neural glial antigen 2 (NG2) (**b**) and alpha smooth muscle actin (α-SMA) (**c**).

To verify the characteristics of PCs used, we investigated the expression of commonly applied PC markers at the transcriptional level [8,15,22] (Figure 4a). Several marker genes were highly expressed in our PCs, including platelet-derived growth factor receptor-beta (PDGFR-β), alpha-smooth muscle actin (ACTA2), vimentin (VIM), regulator of G-protein signaling (RGS5), amino peptidase N (ANPEP) and non-muscle myosins (MYH9/10). To a lower extent also desmin (DES) was expressed in our PCs. Additionally, interferon-induced transmembrane protein 1 (IFITM1), a rather recently identified PC marker [23], was also highly expressed in the used PCs. The expression levels of the commonly applied PC markers nestin (NES), chondroitin sulfate proteoglycan 4 (CSPG4/NG2) and melanoma cell adhesion molecule (MCAM/CD146) were rather low. On the protein level, however, NG2 was detected (Figure 4b). Furthermore, ACTA2, RGS5, desmin, MYH10 and IFITM1 were transcriptionally increased upon co-culture with ECs, whereas the expression of ANPEP was slightly reduced (Supplementary Table S4). Transcriptional levels of the other marker genes, including PDGFR-β, remained unchanged upon co-culture.

### 3.3. Effect of Co-Culture on Proteins of the Extracellular Matrix

Many basic constituents of the extracellular matrix (ECM), including different collagens, integrins and laminins, were up- or downregulated in co-cultured PCs (Supplementary Tables S5 and S6). Additionally, many key remodeling enzymes of the ECM, such as MMPs and ADAM(TS), were differentially expressed in co-cultured PCs. The high impact of co-culture on ECM regulation is also reflected in the results from BioPlanet pathway analysis, with the most highly differentially regulated pathway being ‘TGF-β regulation of extracellular matrix’ (Table 3), but also includes ‘Interleukin-1 regulation of extracellular matrix’. Several ECM genes involved in activation of transforming growth factor beta (TGF-β) are regulated, such as different isoforms of the latent TGF-β-binding protein and the fibulin-3 gene EFEMP1.

### 3.4. Effect of Co-Culture on Inflammatory Profile

Among the most highly regulated genes in PCs co-cultured with ECs, a great number relate to inflammation. This is also evident in the pathway enrichment analysis on DRGs (both BioPlanet and GO Biological Processes), which resulted in a list of pathways with several thereof being linked to inflammatory and viral signaling (Table 3; Supplementary Table S2). A selection of DRGs associated with inflammation is listed in Supplementary Tables S5 and S6. Surprisingly, many chemokine ligands are upregulated in co-cultured PCs, with many of them belonging to the (X-C-X)- motif subfamily. Additionally, CCL-2, CCL-20, CX3CL1 and IL-6 were upregulated upon co-culture. Furthermore, many isotypes of the MHC class 1 genes (HLA) and several interferon-induced genes (IFI(T)) were upregulated. On the other hand, two suppressors of cytokine signaling (SOCS2 and SOCS4) were increased upon co-culture as well.

### 3.5. Effect of Co-Culture on Secreted Soluble Proteins

Next to the cytokines and chemokines, other soluble factors were also changed upon co-culture in PCs. Among them, several paracrine mediators involved in EC–PC crosstalk are included, such as angiopoietin 1, TGF-β -2 and -3, basic fibroblast growth factor (FGF-2) and brain-derived neurotrophic factor (BDNF), which were all upregulated in co-cultured cells (Supplementary Table S5). Transcriptional expression levels of VEGF-A, galectin-3, as well as Wnt 9B and Wnt 5A, were decreased (Supplementary Table S6).

### 3.6. Effect of Co-Culture on Transporters, Carriers, Ion Pumps and G Protein-Coupled Receptors

In PCs co-cultured with ECs vs. PCs cultured alone, many members of the solute carrier families, ATP-binding cassettes, ion transporters and G protein-coupled receptors (GPCRs) were regulated (Supplementary Tables S7 and S8). This includes ion-dependent carriers for amino acids (SLC1A1, SLC7A2, SLC38A1/9), glucose(-derivatives) (SLC2A1, SLC17A5), neurotransmitters (SLC6A6/9), monocarboxylates (SLC16A1/6/7), cholines (SLC44A1/4), protons (SLC9A3/9), phosphates (SLC20A1) and sulfates (SLC26A2). Furthermore, several mitochondrial transporters (members of the SLC25 family) as well as proteins regulating nucleotide sugar transport between the cytosol and Golgi apparatus or endoplasmic reticulum (SLC35 family members) are also differentially regulated. We further noted an upregulation of two potassium channels (KCNJ6, KCNT1), two sodium channels (SCN3A, NALCN), one chloride channel (CLIC4) and three non-selective cation channels (TRPC4/6 and TRPM7).

### 3.7. Effect of Co-Culture on Other Proteins

An interesting finding is the upregulated expression of the connexin 43 gene (GJA1), one of the major gap–junction proteins involved in direct EC–PC contact formation [24]. Furthermore, we observed a reduced expression of two prostaglandin E receptors (EP2 and EP4), ephrin A5, EGFR and two of its ligands (epiregulin and neuregulin), as well as of the SMAD family members 1, 3 and 7. The antioxidative enzymes superoxide dismutase 1 and 2 were transcriptionally increased upon co-culture. Additionally, several TGF-β-induced proteins are upregulated including calpain, smooth muscle gamma actin, tropomyosin and transgelin (Supplementary Tables S5 and S6).

### 3.8. Cytokine Proteome Profiler

**Figure 5:**
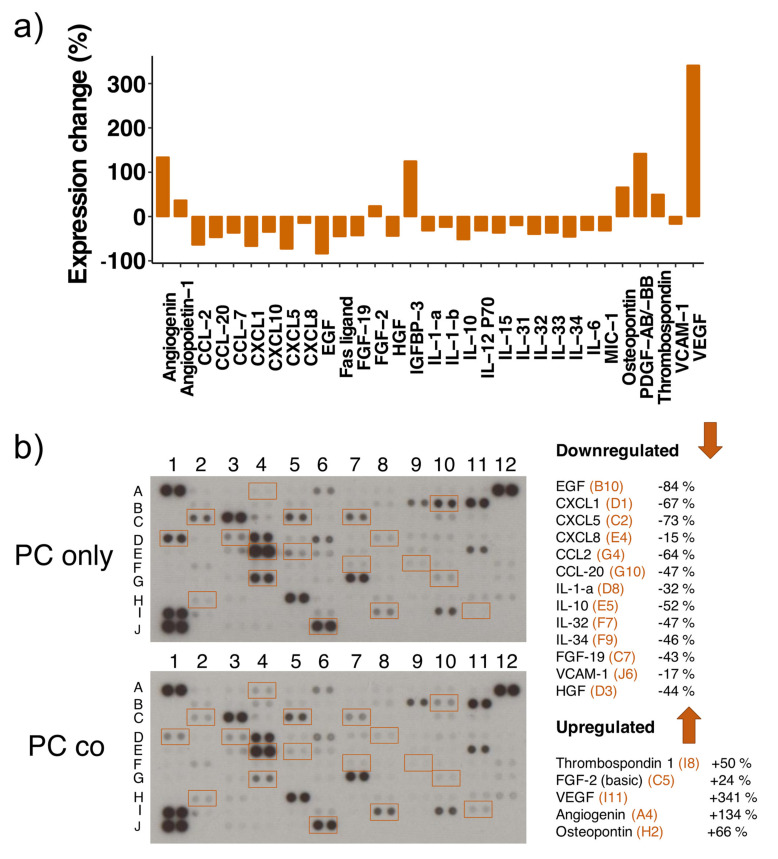
Differential expression of several cytokines in mono- and co-cultured pericytes (PCs). Cytokine proteome analysis has been performed with the Proteome Profiler Human XL Cytokine Array Kit using equal amounts of cell lysates from PCs cultured alone (PC only) and in co-cultures with endothelial cells (PC co). Experiments were performed twice with independently prepared samples. The bar graph shows the changes in protein expression levels in % between co- and mono-cultured PCs (positive values: increased expression in co-culture, negative values: decreased expression in co-culture) (**a**). Included are results from 5- and 10 min array blot exposure and only proteins regulated accordingly in both experiments are shown in the bar graph. Representative array blots are shown after an exposure time of 5 min (**b**). Array blots from both experiments and exposure times are shown in Supplementary Figure S3. The legends on the right of the array blots denote the regulated proteins together with the coordinates for their location on the blots in orange.

Since a great number of DRGs observed in the microarray data are inflammation-related, we performed a cytokine proteome array on cell lysates of mono- and co-cultured PCs. This membrane-based antibody array detects the relative protein expression levels by means of chemiluminescence, and we used two different exposure times (5 min and 10 min) to capture more and less abundantly expressed proteins. To our astonishment, many of the transcriptionally upregulated cytokines were downregulated on the protein level (Figure 5/Table 4). This includes the various C-X-C motif chemokine ligands (−15 to −73%) as well as CCL-2 (−64%), CCL-20 (−47%) and IL-6 (−31%). Furthermore, several cytokines that were not significantly regulated on the transcriptional level showed a decreased protein expression, such as IL-10 (−52%), IL-15 (−37%), IL-12 P70 (−32%), IL-31 to -34 (32-46%), IL-1-α (−32%) and IL-1-β (−24%). Also, the two growth factors HGF and EGF were downregulated, as well as Fas ligand, VCAM-1 (−17%), FGF-19 (−43%) and MIC-1 (−32%). On the other side, VEGF-A expression was increased (+341%) dramatically upon co-culture, which was confirmed by Western blot analysis (Supplementary Figure S2), even though we observed its downregulation on the transcriptional level. Additional upregulated proteins include angiopoietin-1 (+37%), FGF-2 (+24%), thrombospondin-1 (+50%), osteopontin (+66%), PDGF-AB/-BB (+142%) and IGFBP-3 (+125%).

### 3.9. Overlap of Transcriptomic Changes in Co-Cultured Pericytes and Endothelial Cells

**Figure 6:**
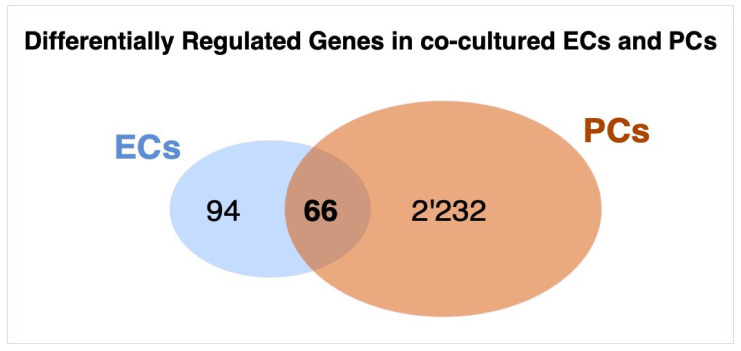
Venn diagram of differentially regulated genes (DRGs) between co-cultured endothelial cells (ECs) and pericytes (PCs). ECs and PCs were cultured alone or together in co-culture on permeable Transwell inserts for 7 days, before they were lysed for RNA extraction and micro-array analysis. A total of 160 DRGs were observed in co- vs. mono-cultured ECs, while co- vs. mono-cultured PCs the number of DRGs was 2298. A total of 66 genes were common in both comparisons. Comparison of gene expression data of co-cultures versus mono-cultures of each cell-type in triplicates was performed by using Transcriptome Analysis Console (TAC, Applied Biosystems). For the analysis, a fold change (FC) cut-off of 1.5 (≈log2FC +/− 0.59) and FDR *p*-Value of 0.05 was applied.

In a previous study we investigated the transcriptomic changes between ECs cultured alone and in presence of PCs in the same co-culture model as was used in the present study [18]. Interestingly, the number of DRGs observed in co-cultured vs. mono-cultured PCs (2’298) was more than ten-fold higher than the number of observed DRGs in ECs (160). By comparing DRGs from co- vs. mono-cultured ECs and PCs, we found an overlap of 66 genes which are regulated in both cell types upon co-culture, as is shown by the Venn-diagram (Figure 6, Supplementary Table S9). Pathway analysis of these common DRGs again showed high impact on the regulation of inflammatory pathways and ECM regulation (Supplementary Table S10).

## 4. Discussion

Since pericyte–endothelial cell (PC–EC) crosstalk essentially contributes to the formation and stabilization of the endothelial barrier, the impact of these mural cells on EC function has been extensively investigated by us [18] and others [2,25,26]. We recently demonstrated that basolateral contact between ECs and PCs regulates multiple genes and cytokines/chemokines in human brain microvascular ECs [18]. As an extension, in the present study we demonstrate that also ECs greatly impact the transcriptomic profile of PCs when the two cell types are co-cultured together on opposite sides of a Transwell insert. To the best of our knowledge, this is the first transcriptomic study conducted so far in PCs using this co-culture model with contact of PCs and ECs through the pores of a Transwell insert. Importantly, this is a fully human model which is commonly used for in vitro BBB studies and our findings provide novel insights into EC–PC crosstalk with the focus on PC function. In the following discussion we look at different aspects of PC function and how the observed transcriptional changes induced by ECs might affect the behavior of these perivascular cells.

### 4.1. PC Markers

The heterogenous nature of PCs is reflected in the difficulties of identifying these cells with respect to sole marker expression. Therefore, PC identity is usually determined by a combination of characteristics such as morphology and anatomical localization together with the expression of not just one, but several protein markers. The PCs used in this study transcriptionally expressed many of the commonly applied PC markers [8,15,22], including platelet-derived growth factor receptor beta (PDGFR-β), alpha-smooth muscle actin, desmin, regulator of G-protein signaling 5 (RGS5), non-muscle myosins, vimentin, amino peptidase and interferon induced transmembrane protein 1. The fact that five out of these highly expressed marker genes are upregulated upon co-culture indicates that the interaction with ECs induces PC maturation. A similar finding was also made by Brandt et al. [22]. The mentioned study investigated the effect of ECs on the PC transcriptome in a direct co-culture model, where the two cell types were plated together in the same dish. Despite the low transcriptional expression of the very commonly used PC marker chondroitin sulfate proteoglycan 2 (NG2), immunofluorescent staining verified its moderate expression on the protein level. NG2/α-SMA co-expression along the microvessel wall has been observed in perivascular cells of capillaries and arterioles, whereas PCs in postcapillary venules were α-SMA-positive but lacked expression of NG2 [27]. The rather low NG2 expression might also indicate that the used PCs (purchased from ScienCell) originate from postcapillary venules rather than arterioles or capillaries.

### 4.2. Inflammatory Profile

Brain PCs are highly versatile with regard to immunological responses and neuroinflammation. On one side, PCs maintain neuronal health by secreting neuroprotective mediators such as brain-derived neurotrophic factor (BDNF) and other pleiotrophins, and by releasing anti-inflammatory signals upon LPS stimulation, thereby contributing to brain homeostasis [11]. On the other side, several recent studies showed pericyte’s potential to react as immune cells in response to inflammatory stimuli such as tumor necrosis factor alpha (TNF-α) or interferon gamma (IFN-γ) by secreting a plethora of chemokines and cytokines [12,15,28]. The high number of inflammatory molecules regulated in PCs co-cultured with ECs reinforce the understanding that PCs are indeed highly susceptible to extracellular impulses regarding alterations in the expression levels of several chemokines and cytokines on the mRNA as well as protein level.

The fact that most of the investigated chemokines and cytokines were downregulated on the protein level upon co-culture suggests that ECs, under basal conditions, exert dampening effects on the inflammatory protein expression of PCs. This is in line with the common understanding that a functional neurovascular unit under homeostatic conditions requires a low inflammatory profile [29]. However, results from the transcriptomic analysis showed just the opposite, with an increase in many chemokine ligands in co-cultured PCs. The contradictory nature of the results between the transcriptomic and proteomic expression of inflammatory mediators is astonishing, however, a similar observation has been made in another study with LPS treated PCs. Despite the transcriptional induction of IL-1-α and IL-1-β, no increase in protein expression was observed [30]. Another report showed a transcriptional upregulation of IL-1-β and TNF-α, but no changes of these molecules on the protein level in human coronary artery ECs [31]. Consistent with our findings from gene expression analysis, Brandt et al. also noted increased transcriptional expression of several chemokines in their comparison between co- and mono-cultured PCs, including CXCL-1, CXCL-3, CXCL-5, CCL-2, IL-1-β and IL-6 [22]. Unfortunately, the protein expression of these molecules was not investigated in this study.

The responsiveness of PCs to inflammatory stimuli occurs rapidly and involves the secretion of different chemokines [28]. For example, PDGFR-β/RGS5 positive perivascular cells release CCL-2 within 2 h of inducing systemic inflammation, while the activation of other cells including astrocytes and microglia occurs at a later stage [32]. Compared to transcriptional control, regulation on the translational level allows for this rapid production of chemokines [33]. Posttranscriptional regulation of cytokines is highly important in coordinating the initiation as well as the termination of an immune response. Adenine and uridine rich sequences (so-called ARE’s) in the 3’ UTR of different chemokine and cytokine mRNAs, including IL-1-β, IL-6, CXCL-8, CCL-2, CCL-20 and IL-10, are associated with their posttranscriptional regulation [34]. Such posttranscriptional control mechanisms of inflammatory mediators include regulation of mRNA nuclear export, mRNA decay or translational repression [33,35]. Pathway analysis of DRGs between co-and mono-cultured PCs showed an enrichment of proteins involved in mRNA processing. This includes many proteins participating in alternative splicing, but also nucleoporins, signal recognition particles and enzymes responsible for mRNA capping and polyadenylation (and hence mRNA stability) are differentially regulated. Translational repression, but not mRNA decay, explains our opposing observation of high mRNA and low protein expression. Since miRNA regulation in animals mostly leads to translational inhibition rather than mRNA degradation [36], posttranscriptional regulation by miRNAs would explain these observations. The increased chemokine mRNA levels in co-cultured PCs might further indicate their intracellular storage, in order to be able to promptly react in case of infection. Processing bodies and stress granules are intracellular assembling units of non-translating mRNAs, which also play an important role in coordinated mRNA storage [37].

Apart from the regulation of different soluble inflammatory mediators, we observed an increased expression of six genes transcribing major histocompatibility (MHC) molecules class I (HLA, human leukocyte antigen). MHC class I proteins present degraded cytosolic proteins to cytotoxic CD8+ T-cells. Under basal conditions, the presented peptides comprise degraded cellular (self) proteins, which are ignored by CD8+ T-cells. Upon infection, the presented peptides contain also fractions of pathogen-derived proteins, which trigger an adaptive immune response. MHC class I molecules also play an important role in innate immunity by serving as inhibitory ligands for natural killer cells, which attack and eliminate cells that lack the expression of self MHC class I proteins [38]. Increased expression of MHC class I molecules in PCs upon co-culture further demonstrates the importance of EC–PC crosstalk on proper immunological function of PCs.

Our finding that ECs dramatically upregulate (2.5 to 8.5 log2 fold) the expression of interferon associated proteins like IFIT1, IFI44L, IFI27, IFIT3, IFI6 and IFI44, together with the fact that these interferon associated proteins restrict replication of BBB disrupting viruses [39,40,41,42,43,44,45,46,47,48,49] suggests that ECs prime the PCs to protect against viral infection induced BBB disruption. Since viral infection could be systemic as well as of brain tissue origin, the PCs are uniquely located to protect against viral insult form both apical and basolateral sides. Interestingly, in contrast to PCs, most of the interferon associated genes were downregulated in ECs co-cultured with PCs (Supplementary Table S11). Although the importance for this differential expression of certain interferon associated genes in ECs and PCs is unclear, it is tempting to speculate that interferon associated proteins may play a selective role in ECs and PCs. Moreover, EC–PC interaction may define the EC driven protective actions of PCs on barrier integrity under pathophysiological conditions.

### 4.3. ECM Proteins

BBB integrity depends not only on paracrine signaling and direct connections between ECs and surrounding cell types, but also relies on the composition of the extracellular matrix (ECM) [9,10]. Interactions of basement membrane proteins with cell surface receptors allow the induction of cellular responses according to the environmental conditions [50]. Both, ECs and PCs, substantially contribute to the deposition of ECM proteins to their common basement membrane. Our results underline the powerful nature of co-culture on the differential expression of basement membrane proteins in PCs. In a recent publication we have demonstrated that the induction of EC barrier function by PCs depends on direct contact through Transwell inserts (Supplementary Figure S4; [18]). The high degree of ECM regulation in PCs co-cultured with ECs shown here represents an explanation for this finding. Indeed, ECs cultured on PC-derived ECM exhibit a tighter barrier function than ECs cultured on their own matrix [51]. Under pathological conditions, where PC migration away from the vessel wall is frequently observed, disruption and/or differential composition of the basement membrane is one cause for the commonly observed barrier dysfunction in different diseases [9].

Pathway analysis revealed a high impact of co-culture on transforming growth factor β (TGF-β) regulation of ECM. It is well-established that co-cultures between ECs and mural cells induces activation of latent TGF-β [52,53]. Ligands of the TGF-β superfamily comprise a large heterogenous group of signaling molecules, including bone morphogenetic proteins (BMPs), activins, inhibins, nodals and several growth and differentiation factors [54]. TGF-β prototypes comprise three different isoforms, all of which are secreted in a latent form, usually bound to latent TGF-β binding proteins (LTBPs). When activated, they can lead to canonical signaling mediated via Smad family member of transcription factors as well as non-canonical signaling [55]. TGF-β signaling therefore strongly depends on the spatial and temporal expression of different activators and repressors. TGF-β target proteins further include many ECM constituents, which themselves are involved in the regulation of TGF-β activation or repression [55]. Due to this high complexity of many involved proteins, a thorough analysis of DRGs of that pathway goes beyond the scope of this study and is omitted.

ECM proteins including multiple integrins, collagens and fibulin-3 play a critical role in maintaining BBB integrity and regulating cell migration, growth and survival. Our finding that in PCs, ECs upregulate fibulin-3 and connective tissue growth factor (CTGF), together with the fact that fibulin-1 and CTGF are essential proteins for BBB genesis and integrity, suggests that ECs can induce pro-BBB actions by influencing PCs [56,57].

ECs also upregulated the expression of integrins, particularly ITGA5 and ITGA8, both of which play a prominent role in maintaining BBB integrity. Selective ablation of α5 integrin leads to cerebral hemorrhage [58], whereas ITGA8 is actively regulated in brain pericytes [59] and regulates microvascular integrity in the kidneys [60]. ITGA8 has been shown to facilitate phagocytosis, adhesion and anti-proliferative as well as anti-migratory actions [61], suggesting that it may prevent PC migration under basal condition and preserve BBB function.

### 4.4. Soluble Factors

Important paracrine signaling factors between PCs and ECs include TGF-β, vascular endothelial growth factor (VEGF), PDGF-B, fibroblast growth factor (FGF) as well as angiopoietin 1 and 2 (Ang1 and 2) [1,4,8]. The observed upregulation of FGF-2 and Ang1 in PC co-cultures on the mRNA as well as on the protein level underlines the importance of these soluble factors in regulating endothelial barrier integrity in EC–PC co-cultures [62,63]. Additionally, we observed a transcriptional decrease of VEGF-A in co-cultured PCs, a finding that was also made by Brandt et al. [22]. On the protein level, however, expression of VEGF-A was highly upregulated in co-cultured PCs, as was determined by the proteome array as well as Western blot analysis. The biological role of VEGF-A is highly complex and depends on concentration and on the presence of VEGF-receptor isoforms on target cells [64]. In this regard, it has been shown that VEGF-A acts as an autocrine inducer of PC proliferation under hypoxic conditions [65]. Furthermore, it has been reported that VEGF-A and FGF-2 increased the stabilization of EC tubes in a 3D in vitro co-culture model with ECs and PCs [66] and Darland et al. have demonstrated that the production of VEGF-A by differentiated PCs in EC–PC co-cultures promotes EC survival and endothelial barrier integrity [67]. On the other hand, VEGF-A is a very potent endothelial mitogen and is induced under hypoxic conditions to increase EC proliferation and angiogenesis, processes that are well-known to exert negative effects on endothelial barrier function [68]. The fact that we previously observed significant improvement of barrier function in our co-culture model (Supplementary Figure S4; [18]) indicates that the observed increase in pericytic VEGF-A expression upon co-culture does not compromise barrier integrity under the used conditions. Since we measured cellular VEGF-A expression, a reduced secretion of the protein by PCs upon co-culture cannot be ruled out. Additionally, our finding that ADAMTS12, a VEGF sequestering metalloproteinase [69], is increased in co-cultured PCs, suggests that ECs keep the BBB disruptive effects of VEGF-A under control.

The probably most prominently investigated pathway regarding PC function is PDGF-B/PDGFR-β pathway, which has been shown to play a critical role in PC recruitment to newly formed vessels as well as in the communication between ECs and PCs [3,4]. The observed increase in PDGF protein levels in co-cultured PCs most probably stems from receptor-bound PDGF that was originally secreted by ECs. Cellular functions of the secreted glycoprotein galectin-3, which was transcriptionally downregulated in co-cultured PCs, are diverse and cell type specific [70]. Next to its angiogenic functions in ECs [71], it has also been shown to exert strong pro-inflammatory actions including superoxide anion production or activation of different cell types involved in the immune response [70]. In this regard, decrease in galectin-3 leads to an overall stabilization of vessel homeostasis. However, this result needs to be confirmed on the protein level in order to make conclusive statements.

### 4.5. Solute Carriers, Transporters, Ion Channels and G Protein-Coupled Receptors (GPCRs)

Pericytes are the first cells to relax during neural activity, leading to capillary dilation and increased cerebral blood flow (CBF) to boost energy supply [72]. Since local ion concentrations substantially contribute to the establishment of resting and action potentials, thereby regulating cell excitability [73,74], the regulation of many ion channels and GPCRs in PCs co-cultured with ECs indicates that co-culture substantially influences the ability of PCs to regulate CBF. Pericyte membrane hyperpolarization has further been implicated to lead to long-range electrical signaling to vascular smooth muscle cells, which further impacts CBF [73].

Solute carriers are important in regulating the transport of a variety of molecules across the BBB and their expression plays a crucial role in maintaining CNS homeostasis as well as in regulating drug delivery into the brain [75]. While SLC expression and function in astrocytes and neurons has been investigated in several studies, the role of carrier-mediated transport in PCs remains largely unexplored. The observed regulation of a multitude of different SLCs in co-cultured PCs suggests that, next to ECs, also PCs might contribute to the active transport of different molecules and metabolites from the blood into the brain and vice versa.

### 4.6. Other Proteins

Crosstalk between ECs and PCs not only relies on secreted paracrine factors, but also on the formation of adhesion plaques and gap junctions, which allow the direct exchange of second messengers, ions and other small molecules between adjacent cells, and play an important role in integrating cellular signals to regulate vascular tone [8]. Interestingly, we observed the transcriptional upregulation of a major protein involved in these direct interactions between ECs and PCs: connexin 43 (cx43) [76]. The significance of cx43 is demonstrated by detrimental effects on barrier integrity observed in an in vivo study with cx43 knock out mice [77]. Its abnormal expression in diabetes is further associated with the development of different complications such as the vasomotor decline observed in diabetic retinopathy [78]. Interestingly, gap junction communication is required for endothelial-induced mural cell differentiation. Mural cell cx43 expression is necessary for mediating TGF-β activation, which leads to transcriptional upregulation of different mural cell specific genes [79]. Since cx43 as well as several of TGF-β-induced mural cell specific genes, such as smooth muscle α- and γ-actin, caldesmon, tropomyosin 1, SM22-α and SM myosin heavy chain 11, are profoundly upregulated in co-cultured PCs, our results indirectly show TGF-β activation upon co-culture does indeed take place in PCs. Additionally, the expression of contractile molecules such as smooth muscle actins and myosins underlines the ability of PCs to modulate vascular tone and cerebral blood flow. Our finding that ECs downregulate prostaglandin receptor 2 (EP2) expression in PCs, together with the fact that COX-2 derived prostaglandins disrupt BBB and EP2 receptor blockers reduce delayed mortality and brain inflammation in status epilepticus [80], suggests that under basal conditions ECs upregulate anti-inflammatory mechanisms capable to disrupting BBB. Apart from COX-2, PGE2 signaling may also influence other mechanisms. In a detailed study, Perrot et al. linked prostaglandin E2 (PGE2) signaling to cx43 (and N-cadherin) downregulation in PCs via Ca^2+^-induced calpain, eventually leading to vessel destabilization [76]. The observed decrease of PGE2 receptors 2 and 4, as well as calpain-2 upon co-culture in our microarray analysis might therefore be related to the increase in cx43 levels.

EGF ligands are transmembrane proteins with an extracellular receptor binding domain and proteolytical cleavage of this EGF motif releases a soluble growth factor [81]. This so-called ectodomain shedding might explain the observed decrease in EGF protein levels in co-cultured PCs, which is also supported by the transcriptional increase of ADAM9 and ADAM10, two main metalloproteinases responsible for shedding of EGFR ligands [81]. The expression of EGFR has been shown to be localized at PC–EC interdigitations in immature capillaries on the pericytic cell membrane, which was completely lost upon capillary maturation [82]. This finding suggests that the reduced expression of EGFR and two of its ligands, epiregulin and neuregulin 1, in co-cultured PCs is an indication for pericyte/vessel maturation. Furthermore, the expression of insulin-like binding protein-3 (IGFBP-3) has been linked to endothelial nitric oxide production and vascular and neuronal protection after ischemic injury in the retina, while loss of IGFBP-3 is associated with increased TNF-α levels [83,84,85,86]. Increased protein expression of IGFBP-3 in co-cultured PCs is yet a further demonstration for the shift towards a neuroprotective phenotype upon their close and physiological interaction with ECs.

The superoxide radical is one of the main reactive oxygen species in the vasculature and can severely affect vascular function. It is both a cause and a consequence of vascular dysfunction and is related to many neuro- and cardiovascular disorders [87]. Its neutralization by superoxide dismutase (SOD) enzymes is a critical step in the antioxidant defense. The fact that two out of three SOD isoforms are transcriptionally upregulated in co-cultured PCs suggests that ECs potentiate the neutralization of superoxide radicals in PCs.

A limitation of the present study is the use of an endothelial cell line. It would be important to confirm our results with primary human brain microvascular ECs. Furthermore, our in vitro findings need to be validated in an in vivo (-like) setting. Additionally, the heterogenous nature of pericytes [8] should also be taken into account, even though the pericytes used in this study expressed pericyte marker genes like PDGFR-β, ANPEP, Vimentin, RGS5 and ACTA2.

## 5. Conclusions

Taken together, the present study substantiates the importance of endothelial cell—pericyte crosstalk and highlights the impact of endothelial cells on the pericyte transcriptomic profile. To the best of our knowledge, this is the first transcriptomic study investigating the impact of co-culturing ECs and PCs on opposite sides of a Transwell membrane on the gene expression in PCs. Our results further highlight the multipotency of pericyte action in the vasculature by disclosing the co-culture-induced upregulation of molecules involved in BBB establishment and CNS homeostasis as well as in the regulation of cerebral blood flow. We further show that co-culture induces significant changes in the inflammatory profile of PCs. While chronic inflammation is associated with detrimental effects on brain function and is the most common underlying cause for many cardiovascular and neurological diseases such as Alzheimer’s disease or multiple sclerosis, the transient and controlled neuro-inflammation is an essential step for tissue repair and for the restoration of homeostasis after infection or disease [88]. This initial induction of cytokines and chemokines is mediated by a temporary activation of glial cells, astrocytes and macrophages [89]. The presented results provide first evidence that endothelial cells induce a downregulation of the pro-inflammatory profile in pericytes on the protein level, while transcriptomic expression of the same inflammatory mediators is enhanced. These observations indicate that pericytes, by rapid translational rather than transcriptional control, are able to promptly react to inflammatory stimuli, while they maintain a neuroprotective phenotype under basal conditions. In this way, our findings reinforce recent reports that pericytes contribute to the initial inflammatory response in the CNS. Upregulation of interferons and genes induced by these cytokines in pericytes may also play a role in protecting EC damage and barrier dysfunction induced by viruses, including SARS-CoV-2 [90]. Interestingly, in critically ill COVID-19 patients, damage to pulmonary pericytes [91] as well as a diminished or delayed interferon response is observed [90]. Based on these observations, it is tempting to speculate that severe COVID-19 infection may directly or indirectly be induced by altered EC–PC dynamics that are linked to compromised interferon generating pathways and dysfunctional EC barrier integrity.

## Figures and Tables

**Table 1:** Top ten upregulated genes in co-cultured pericytes.

Gene	Gene Description	Log2 FC (Co- Vs. Mono-Culture)	FDR *p*-Value
IFIT1	Interferon-induced protein with tetratricopeptide repeats 1	8.5	1.59 × 10^−16^
MX1	MX dynamin-like GTPase 1 (interferon-induced)	7.3	7.05 × 10^−15^
IFI44L	Interferon-induced protein 44-like	6.2	1.80 × 10^−13^
CXCL8	Chemokine (C-X-C motif) ligand 8	5.8	3.89 × 10^−12^
IFI6	Interferon, alpha-inducible protein 6	5.2	1.30 × 10^−9^
XAF1	XIAP associated factor 1	5.2	1.58 × 10^−13^
SAMD9L	Sterile alpha motif domain containing 9-like	5.2	1.61 × 10^−9^
SAMD9	Sterile alpha motif domain containing 9	5.2	2.18 × 10^−11^
OAS1	2-5-oligoadenylate synthetase 1	5.1	4.12 × 10^−14^
RCAN1	Regulator of calcineurin 1	4.8	4.36 × 10^−13^

Log2 fold changes (log2FC) and adjusted P-values are depicted in the third and fourth column, respectively. Comparison of gene expression data of co-culture vs. mono-culture of PCs in triplicates was performed by using Transcriptome Analysis Console (TAC, Applied Biosystems). For the analysis a fold change (FC) cut-off of 1.5 (≈log2FC +/− 0.59) and FDR *p*-Value of 0.05 was applied.

**Table 2:** Top ten downregulated genes in co-cultured pericytes.

Gene	Gene Description	Log2 FC (Co- Vs. Mono-Culture)	FDR *p*-Value
NPTX1	Neuronal pentraxin I	−5.2	4.12 × 10^−14^
JUN	Jun proto-oncogene	−3.6	2.96 × 10^−9^
SLC6A6	Solute carrier family 6 (neurotransmitter transporter), member 6	−3.5	1.28 × 10^−8^
PTGER2	Prostaglandin E receptor 2	−3.5	2.50 × 10^−7^
MN1	Meningioma (disrupted in balanced translocation) 1	−3.5	6.56 × 10^−8^
F2RL2	Coagulation factor II (thrombin) receptor-like 2	−3.4	7.00 × 10^−7^
SLC14A1	Solute carrier family 14 (urea transporter), member 1 (Kidd blood group)	−3.3	8.03 × 10^−8^
STEAP3	STEAP family member 3, metalloreductase	−3.0	3.05 × 10^−7^
SCARA3	Scavenger receptor class A, member 3	−3.0	4.89 × 10^−6^
SHISA2	Shisa family member 2	−2.8	0.0055

Log2 Fold Changes (log2FC) and adjusted P-values are depicted in the third and fourth column, respectively. Comparison of gene expression data of co-culture vs. mono-culture of PCs in triplicates was performed by using Transcriptome Analysis Console (TAC, Applied Biosystems). For the analysis a fold change (FC) cut-off of 1.5 (≈log2FC +/− 0.59) and FDR *p*-Value of 0.05 was applied.

**Table 3:** Pathway enrichment analysis (BioPlanet) of differentially regulated genes (DRGs) between pericytes cultured with and without endothelial cells (ECs).

Pathway	Overlap	Adj. *p*-Value
TGF-beta regulation of extracellular matrix	132/565	1.09 × 10^−11^
Epidermal growth factor receptor 1 (EGFR1) pathway	45/152	2.32 × 10^−6^
Brain-derived neurotrophic factor (BDNF) signaling pathway	63/261	8.33 × 10^−6^
T cell receptor regulation of apoptosis	118/603	8.33 × 10^−6^
Follicle-stimulating hormone (FSH) regulation of apoptosis	63/263	8.77 × 10^−6^
Messenger RNA processing	52/203	1.14 × 10^−5^
Interleukin-1 regulation of extracellular matrix	36/120	1.64 × 10^−5^
Interferon alpha/beta signaling	24/64	2.03 × 10^−5^
Smooth muscle contraction	13/22	2.41 × 10^−5^
Interferon signaling	44/168	3.61 × 10^−5^
Interleukin-2 signaling pathway	149/847	5.46 × 10^−5^
Ataxia telangiectasia mutated (ATM) pathway	19/48	1.05 × 10^−4^
Interleukin-4 regulation of apoptosis	58/267	3.54 × 10^−4^
Lipid metabolism regulation by peroxisome proliferator-activated receptor alpha (PPAR-alpha)	31/112	4.19 × 10^−4^
Lipid and lipoprotein metabolism	92/489	4.28 × 10^−4^
Fatty acid, triacylglycerol, and ketone body metabolism	41/173	8.60 × 10^−4^
Activator protein 1 (AP-1) transcription factor network	22/70	9.30 × 10^−4^
Integrated breast cancer pathway	37/152	1.07 × 10^−3^
Retinoic acid receptor-mediated signaling	13/30	1.09 × 10^−3^
Gene expression	158/968	1.17 × 10^−3^
Angiogenesis	11/23	1.34 × 10^−3^
Senescence and autophagy	27/99	1.34 × 10^−3^
Uridine diphosphate (UDP)-N-acetyl-glucosamine biosynthesis	5/5	1.34 × 10^−3^
Capped intron-containing pre-mRNA processing	34/138	1.34 × 10^−3^
Type II interferon signaling (interferon-gamma)	17/50	1.91 × 10^−3^

Pathway enrichment analysis of differentially regulated genes (DRGs) between pericytes cultured with and without endothelial cells. Analysis was performed using NCATS BioPlanet on the Enrichr website by uploading DRGs as determined by the Transcriptome Analysis Console (TAC). Second column describes number of regulated genes compared to total number of genes in the pathway, and p-values adjusted for multiple testing are denoted in the last column. The 25 most significant pathways are listed.

**Table 4:** List of differentially expressed proteins using a cytokine proteome array.

Protein	Array Coordinates	Protein Regulation	mRNA Regulation
CCL2	G4	−64%	up
CCL7	G5	−37%	down (ns)
CCL20	G10	−47%	up
CXCL1	D1	−67%	up
CXCL5	C2	−73%	up
CXCL8	E4	−15%	up
CXCL10	F10	−35%	up
EGF	B10	−84%	nr
Fas ligand	C4	−45%	nr
FGF-19	C7	−43%	nr
HGF	D3	−44%	nr
IL-1-a	D8	−32%	nr
IL-1-b	D9	−24%	up (ns)
IL-6	E3	−31%	up
IL-10	E5	−52%	nr
IL-15	E9	−37%	nr
IL-31	F6	−20%	nr
IL-32	F7	−40%	nr
IL-33	F8	−37%	nr
IL-34	F9	−46%	down (ns)
IL-12 P70	E7	−32%	nr
MIC-1	C10	−32%	nr
VCAM1	J6	−17%	up
Angiogenin	A4	+134%	nr
Angiopoietin 1	A5	+37%	up
FGF-2	C5	+24%	up
IGFBP 3	D7	+125%	down
Osteopontin	H2	+66%	nr
PDGF-AB/-BB	H4	+142%	nr
Thrombospondin-1	I8	+50%	nr
VEGF-A	I11	+341%	down

List of differentially expressed proteins using a cytokine proteome array (membrane-based, chemiluminescent detection). Two different exposure times were applied, and array coordinates are shown in the second column for localization of spots in Figure 5b/Supplementary Figure S3. Column three denotes the observed change in protein expression in co-cultures in relation to protein levels in mono-cultures. Analysis was performed with the image processing software ImageJ (including background subtraction). The last column denotes regulation of the corresponding mRNA levels upon co-culture, as was determined by the microarray (upregulated (up); downregulated (down); not regulated (nr); ns: non-significantly with a *p*-Value < 0.05, but FDR *p*-Value > 0.05).

## Data Availability

All data supporting the findings of this study are available within the article and its supplemental data file or from the corresponding author upon reasonable request.

## Supplementary Materials

The following are available online at https://www.mdpi.com/article/10.3390/cells10071784/s1, Figure S1: Principle Component Analysis, Figure S2: VEGF-A expression in mono- and co-culture pericytes, Figure S3: Array blots from Proteome Profiler Human XL Cytokine Array Kit, Figure S4: Measurements of endothelial barrier function with and without pericytes (PCs) in different constellations (recently published in *Cells* [18]), Table S1: List of highly regulated genes as confirmed by qRT-PCR, Table S2: Pathway enrichment analysis (GO biological processes), Table S3: Pathway enrichment analysis (KEGG), Table S4: List of pericyte marker gene expression significantly regulated upon co-culture with ECs, Table S5: Selection of upregulated genes in co- vs. mono-cultured pericytes, Table S6: Selection of downregulated genes in co- vs. mono-cultured pericytes, Table S7: List of upregulated transporters, carriers, ion pumps and G protein-coupled receptors in pericytes co-cultured with endothelial cells vs. pericytes cultured alone, Table S8: List of downregulated transporters, carriers, ion pumps and G protein-coupled receptors in pericytes co-cultured with endothelial cells vs. pericytes cultured alone, Table S9: Differentially regulated genes (DRGs) that are common in endothelial cells (ECs) and pericytes (PCs) upon co-culture, Table S10: Pathway enrichment analysis (BioPlanet)of differentially regulated genes (DRGs) that are common in ECs and PCs, Table S11: Differential regulation of interferon-induced genes in co-cultured PCs and ECs.
