# Supplementary material for: Transcryptomic Analysis of Human Brain -Microvascular Endothelial Cell Driven Changes in -Vascular Pericytes
Source: Cells. 2021 Jul 14;10(7):1784. doi: 10.3390/cells10071784 (PMC8304094; doi:10.3390/cells10071784)
Supplement: Supplementary file 1 [file cells-10-01784-s001.zip › cells-1221296-supplementary.pdf]

Supplementary Material/data

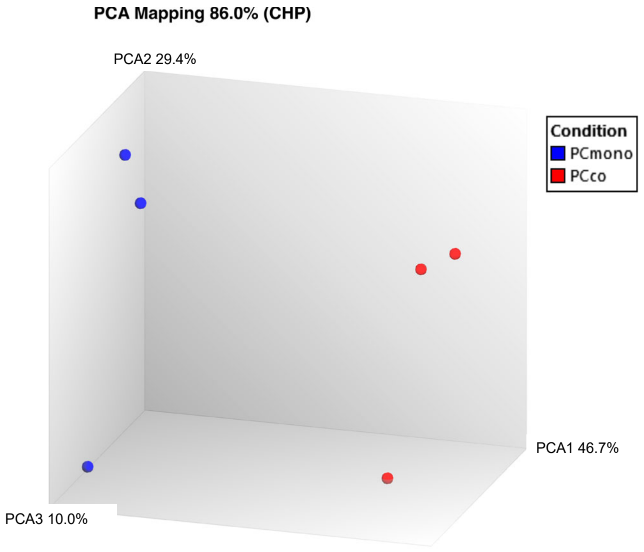

Figure S1: Principle Component Analysis (PCA).

PCA shows a clear separation between the samples from pericyte mono cultures (blue) and co-cultures (red) **(a)**. PCA was automatically performed by the Transcriptome Analysis Console (TAC).

Table S1: List of highly regulated genes as confirmed by qRT-PCR.

Gene	Gene description	rel. expression level (%) upon co-culture	Cat. No.
IFIT1	Interferon-induced protein with tetratricopeptide repeats 1	241 (+/- 49)	PPH01332F
MX1	MX dynamin-like GTPase 1 (interferon-induced)	209 (+/- 10)	PPH01325A
IFI44L	Interferon-induced protein 44-like	362 (+/- 25)	PPH05811G
IFI6	Interferon, alpha-inducible protein 6	209 (+/- 2)	PPH01322C
XAF1	XIAP associated factor 1	141 (+/- 25)	PPH58211A
NPTX1	Neuronal pentraxin I	76 (+/- 23)	PPH10301B
JUN	Jun proto-oncogene	38 (+/- 6)	PPH00095A
SLC6A6	Solute carrier family 6 (neurotransmitter transporter), member 6	67 (+/- 10)	PPH01451E
GAPDH	Glyceralaldehyde-3-phosphate dehydrogenase	-	PPH00150F
PDGFRB	Platelet-derived growth factor receptor-beta	-	PPH00477C

For confirming microarray results, qRT-PCR has been performed using the Custom RT2 PCR arrays from Qiagen in a 96-wellplate format. The tested genes are listed together with their relative expression levels (co-cultured vs. mono-cultured cells) in the third culumn. The catalogue number

(cat.no.) is noted in the last column. The experiment has been performed once in triplicates and expression levels represent mean (+/- standard deviation of the mean).

Table S2: Pathway enrichment analysis (GO biological processes) of differentially regulated genes (DRGs) between pericytes cultured with and without endothelial cells (ECs).

Pathway	Overlap	adj. P-value
Positive Regulation of gene expression	152/771	5.70E-07
Type I interferon signaling pathway	26/65	1.07E-05
mRNA processing	67/283	1.72E-05
Positive regulation of gene expression, epigenetic	21/47	1.72E-05
Positive regulation of transcription, DNA-templated	192/1120	4.79E-05
Regulation of transcription, DNA-templated	255/1598	1.75E-04
Regulation of transcription from RNA polymerase II promoter	237/1478	2.86E-04
Transcription, DNA-templated	74/356	4.74E-04
Regulation of cyclin-dependent protein serine/threonine kinase activity	23/67	5.88E-04
Regulation of viral genome replication	22/63	6.50E-04
Regulation of gene expression, epigenetic	24/74	9.27E-04
RNA splicing, via transesterification reactions with bulged adenosine as nucleophile	53/236	1.06E-03
mRNA splicing, via spliceosome	57/261	1.08E-03
mRNA metabolic process	28/97	1.49E-03
Transcription from RNA polymerase II promoter	91/485	1.63E-03
Regulation of keratinocyte differentiation	13/29	2.27E-03
Cellular response to cadmium ion	12/29	2.27E-03
Positive regulation of macromolecule metabolic process	58/276	2.27E-03
Negative regulation of apoptotic process	90/485	2.30E-03
Positive regulation of keratinocyte differentiation	8/12	2.59E-03
Regulation of gene expression	168/1037	2.68E-03
Regulation of alcohol biosynthetic process	14/34	2.68E-03
Embryonic limb morphogenesis	14/34	2.68E-03
Positive regulation of transcription from RNA polymerase II promoter	141/848	3.38E-03
Cellular response to oxidative stress	30/115	3.63E-03

Pathway enrichment analysis of differentially regulated genes (DRGs) between pericytes cultured with and without endothelial cells. Analysis was performed using GO Biological Processes on the Enrichr website by uploading DRGs as determined by the Transcriptome Analysis Console (TAC). Second column describes number of regulated genes compared to total number of genes in the pathway, and p-values adjusted for multiple testing are denoted in the last column. The 25 most significant pathways are listed.

Supplementary Table S3: Pathway enrichment analysis (KEGG) of differentially regulated genes (DRGs) between pericytes cultured with and without endothelial cells (ECs).

Pathway	Overlap	adj. P-value
Cellular senescence	39/156	1.09E-03
Mitophagy	22/68	1.09E-03
Axon guidance	41/182	3.77E-03
Influenza A	39/172	3.77E-03
Kaposi sarcoma-associated herpesvirus infection	42/193	4.04E-03
Human cytomegalovirus infection	47/225	4.04E-03
Regulation of actin cytoskeleton	45/218	6.49E-03
IL-17 signaling pathway	24/94	7.87E-03
Hepatocellular carcinoma	36/168	1.05E-02
Proteoglycans in cancer	41/205	1.73E-02
Biosynthesis of unsaturated fatty acids	10/27	1.95E-02
Pathways in cancer	88/531	1.97E-02
Colorectal cancer	21/86	2.22E-02
Oxitocin signaling pathway	32/154	2.32E-02
Rheumatoid arthritis	22/93	2.32E-02
Transforming growth factor (TGF) -beta signaling pathway	22/94	2.50E-02
Autophagy	29/137	2.50E-02
Epstein-Barr virus infection	39/202	2.58E-02
Measles	29/139	2.64E-02
Protein processing in endoplasmic reticulum	34/171	2.64E-02
Vascular smooth muscle contraction	28/133	2.64E-02
Prostate cancer	22/97	2.72E-02
Transcriptional misregulation in cancer	37/192	2.72E-02
Human T-cell leukemia virus 1 infection	41/219	2.72E-02
Legionellosis	15/57	2.72E-02

Pathway enrichment analysis of differentially regulated genes (DRGs) between pericytes cultured with and without endothelial cells. Analysis for KEGG pathways was performed on the Enrichr website by uploading DRGs as determined by the Transcriptome Analysis Console (TAC). Second column describes number of regulated genes compared to total number of genes in the pathway, and p-values adjusted for multiple testing are denoted in the last column. The 25 most significant pathways are listed.

Table S4: List of pericyte marker gene expression significantly regulated upon co-culture with ECs.

Gene	Gene description	log2 FC (co- vs. mono-culture)	FDR p-value
ACTA2	Actin, alpha 2, smooth muscle, aorta	4.0	1.58E-13
IFITM1	Interferon induced transmembrane protein 1	6.2	3.72E-11
RGS5	Regulator of G-protein signaling 5	1.4	0.0005
MYH10	Myosin, heavy chain 10, non-muscle	1.9	0.0007
DES	Desmin	2.1	0.004

ANPEP	Alanyl (membrane) aminopeptidase	-1.2	0.0008

For differentially regulated genes the log2 fold change (log2 FC) together with the false discovery rate p-value (FDR p-value) is noted in the third and fourth column, respectively. For the analysis a fold change (FC) cut-off of 1.5 (≈log2FC +/- 0.59) and FDR p-value of 0.05 was applied.

Table S5: Selection of upregulated genes in co- vs. mono-cultured pericytes (PCs).

Gene	Gene description	log2 FC (co- vs. mono-culture)	FDR p-value
**ECM**			
COL11A1	Collagen, type XI, alpha 1	1.9	7.85E-05
COL21A1	Collagen, type XXI, alpha 1	1.6	0.0005
COL8A1	Collagen, type VIII, alpha 1	2.1	0.0008
COL12A1	Collagen, type XII, alpha 1	1.1	0.036
ITGAV	Integrin alpha V	2.1	2.89E-05
ITGA8	Integrin alpha 8	3.3	5.40E-08
EFEMP1	EGF containing fibulin-like ECM protein1 (fibulin-3)	2.5	3.53E-08
CTGF	Connective tissue growth factor	1.7	0.036
ADAM9	ADAM metallopeptidase domain 9	0.8	0.036
ADAM10	ADAM metallopeptidase domain 10	0.8	0.049
ADAMTS5	ADAM metallopeptidase with thrombospondin type 1 motif 5	1.6	2.29E-06
ADAMTS12	ADAM metallopeptidase with thrombospondin type 1 motif 12	2.2	0.0044
**Inflammatory genes**			
CXCL1	Chemokine (C-X-C motif) ligand 1	2.6	6.71E-06
CXCL8	Chemokine (C-X-C motif) ligand 8	5.8	3.89E-12
CXCL5	Chemokine (C-X-C motif) ligand 5	1.1	0.0323
CXCL6	Chemokine (C-X-C motif) ligand 6	1.6	0.0023
CXCL10	Chemokine (C-X-C motif) ligand 10	1.2	0.0148
CCL2	Chemokine (C-C motif) ligand 2	3.0	7.26E-09
CCL20	Chemokine (C-C motif) ligand 20	4.3	2.71E-09
CX3CL1	Chemokine (C-X3-C motif) ligand 1	3.6	2.86E-06
IL6	Interleukin 6	2.3	4.25E-07
SOCS4	Suppressor of cytokine signaling 4	1.7	0.0274
SOCS2	Suppressor of cytokine signaling 2	0.9	0.0125
ALCAM	Activated leukocyte adhesion molecule	2.5	6.68E-06
NCAM2	Neural cell adhesion molecule 2	1.8	0.0005
VCAM1	Vascular cell adhesion Molecule 1	1.3	0.0101
TLR4	Toll-like receptor 4	1.4	5.69E-05
TLR3	Toll-like receptor 3	1.8	1.44E-06

IFIT1	Interferon-induced protein with tetratricopeptide repeats 1	8.5	1.59E-16
IFI44L	Interferon-induced protein 44-like	6.2	1.80E-13
IFI27	Interferon, alpha-inducible protein 27	4.0	1.09E-11
IFIT3	Interferon induced protein with tetratricopeptide repeats 3	3.0	3.72E-11
IFI6	Interferon, alpha-inducible protein 6	5.2	1.30E-09
IFI44	Interferon-induced protein 44	2.5	1.50E-07
IFIH1	Interferon induced, with helicase C domain 1	3.0	2.83E-07
IFIT5	Interferon-induced protein with tetratricopeptide repeats 5	2.0	3.51E-06
IFIT2	Interferon-induced protein with tetratricopeptide repeats 2	2.0	1.05E-05
IFI16	Interferon, gamma-inducible protein 16	1.5	0.0001
IFITM3	Interferon induced transmembrane protein 3	1.2	0.0018
IFI35	Interferon-induced protein 35	0.9	0.0353
HLA-A	Major histocompatibility complex, class I, A	0.9	0.0392
HLA-B	Major histocompatibility complex, class I, B	2.1	1.08E-05
HLA-C	Major histocompatibility complex, class I, C	1.5	0.0037
HLA-F	Major histocompatibility complex, class I, F	1.6	0.0002
HLA-G	Major histocompatibility complex, class I, G	0.6	0.0166
HLA-L	Major histocompatibility complex, class I, L (pseudegene)	1.2	0.0031
**Secreted factors**			
ANGPT1	Angiopoietin 1	1.3	0.0203
ANGPTL	Angiopoietin like 1	1.1	0.0275
FGF2	Fibroblast growth factor 2 (basic)	1.5	0.0005
TGFB2	Transforming growth factor beta 2	1.2	0.006
TGFB3	Transforming growth factor beta 3	2.4	1.05E-05
BDNF	Brain-derived neurotrophic factor	0.6	0.0212
**Other proteins**			
GJA1	Gap junction protein alpha 1 (Connexin43)	1.1	0.0004
ACTG2	Actin, gamma 2, smooth muscle, enteric	4.3	5.83E-09
TPM1	Tropomyosin 1 (alpha)	2.2	7.67E-08
TAGLN	Transgelin (SM22-alpha)	2.0	3.38E-05
MYH11	Myosin, heavy chain 11, smooth muscle	1.2	0.0079
SOD1	Superoxide dismutase 1, soluble	0.7	0.0335
SOD2	Superoxide dismutase 2, mitochondrial	1.4	0.0001

Selection of upregulated genes in co- vs. mono-cultured pericytes (PCs). Log2 fold changes (log2 FC) and adjusted P-values (FDR p-value) are depicted in the third and fourth column, respectively. Comparison of gene expression data of co-culture vs. mono-culture of PCs in triplicates was performed by using Transcriptome Analysis Console (TAC, Applied Biosystems). For the analysis a fold change (FC) cut-off of 1.5 (≈log2FC +/- 0.59) and FDR p-value of 0.05 was applied.

Table S6: Selection of downregulated genes in co- vs. mono-cultured pericytes (PCs).

**Gene**	**Gene description**	**log2 FC (co- vs. mono-culture)**	**FDR p-value**
**ECM**			
COL14A1	Collagen, type XIV, alpha 1	-2.0	1.05E-05
COL6A1	Collagen, type VI, alpha 1	-1.3	0.040
COL6A2	Collagen, type VI, alpha 2	-1.2	0.0093
COL6A3	Collagen, type VI, alpha 3	-1.4	0.0086
ITGA10	Integrin alpha 10	-1.3	0.0009
ITGB4	Integrin beta 4	-1.1	0.011
NID1	Nidogen1	-1.9	1.94E-05
LAMA5	Laminin, alpha 5	-1.3	3.42E-05
LAMC1	Laminin, gamma 1	-0.8	0.037
MMP1	Matrix metallopeptidase 1	-2.5	1.23E-06
MMP2	Matrix metallopeptidase 2	-1.6	0.0064
MMP19	Matrix metallopeptidase 19	-0.85	0.023
ADAMTS15	ADAM metallopeptidase with thrombospondin type 1 motif 15	-2.5	1.56E-06
ADAMTS9	ADAM metallopeptidase with thrombospondin type 1 motif 9	-1.3	0.0008
TIMP1	TIMP metallopeptidase inhibitor 1	-0.9	0.0246
TIMP2	TIMP metallopeptidase inhibitor 2	-0.8	0.0125
TIMP3	TIMP metallopeptidase inhibitor 3	-0.7	0.0023
TGFBI	Transforming growth factor, beta-induced, 68 kDa	-1.2	0.0051
LTBP1	Latent transforming growth factor beta binding protein 1	-1	0.0037
LTBP3	Latent transforming growth factor beta binding protein 3	-1.6	0.0003
LTBP4	Latent transforming growth factor beta binding protein 4	-0.95	0.0209
**Inflammatory genes**			
CXCL12	Chemokine (C-X-C motif) ligand 12	-1.0	0.0004

**Secreted factors**			
ANGPTL4	Angiopoietin like 4	-1.1	0.0416
VEGFA	Vascular endothelial growth factor A	-1.6	8.77E-05
WNT9B	Wingless-type MMTV integration site family, member 9B	-1.3	0.0002
WNT5A	Wingless-type MMTV integration site family, member 5A	-1.2	0.0012
LGALS3	Lectin, galactoside-binding, soluble 3	-1.8	0.0055
**Other proteins**			
PTGER2	Prostaglandin E receptor 2 (EP2)	-3.5	2.50E-07
PTGER4	Prostaglandin E receptor 4 (EP4)	-1.1	0.0223
EGFR	Epidermal growth factor receptor	-0.7	0.0405
LRP1	LDL receptor related protein 1	-2.5	2.85E-06
LRP10	LDL receptor related protein 10	-1.3	0.0246
EREG	Epiregulin	-1.0	0.0213
NRG1	Neuregulin 1	-1.9	0.0002
CAPN2	Calpain (m/II) large subunit	-1.3	0.0201
EFNA5	Ephrin A5	-2-2	1.13E-06
SMAD1	SMAD family member 1	-1.1	0.0033
SMAD3	SMAD family member 3	-2.3	4.49E-07
SMAD7	SMAD family member 7	-1.2	0.0323

Selection of downregulated genes in co- vs. mono-cultured pericytes (PCs). Log2 fold changes (log2 FC) and adjusted P-values (FDR p-value) are depicted in the third and fourth column, respectively. Comparison of gene expression data of co-culture vs. mono-culture of PCs in triplicates was performed by using Transcriptome Analysis Console (TAC, Applied Biosystems). For the analysis a fold change (FC) cut-off of 1.5 (≈log2FC +/- 0.59) and FDR p-value of 0.05 was applied.

Table S7: List of upregulated transporters, carriers, ion pumps and G protein-coupled receptors in pericytes co-cultured with endothelial cells vs. pericytes cultured alone.

Gene	Gene description	log2 FC (co- vs. mono-culture)	FDR p-value
SLC1A1	Solute carrier family 1 (neuronal/epithelial high affinity glutamate transporter, system Xag), member 1	1.5	3.88E-05
SLC4A4	Solute carrier family 4 (sodium bicarbonate cotransporter), member 4	1.8	0.0006
SLC7A2	Solute carrier family 7 (cationic amino acid transporter, y+ system), member 2	0.9	0.0085
SLC16A1	Solute carrier family 16 (monocarboxylate transporter), member 1/MCT1	1.1	0.0011

SLC16A7	Solute carrier family 16 (monocarboxylate transporter), member 7	2.1	0.0011
SLC17A5	Solute carrier family 17 (acidic sugar transporter), member 5	0.8	0.0256
SLC25A13	Solute carrier family 25 (aspartate/glutamate carrier), member 13	0.9	0.013
SLC25A32	Solute carrier family 25 (mitochondrial folate carrier), member 32	1.2	0.0003
SLC25A46	Solute carrier family 25, member 46	1.6	0.0011
SLC26A2	Solute carrier family 26 (anion exchanger), member 2	1.2	0.0034
SLC35A3	Solute carrier family 35 (UDP-N-acetylglucosamine (UDP-GlcNAc) transporter), member A3	1.8	0.0001
SLC35B3	Solute carrier family 35 (adenosine 3-phospho 5-phosphosulfate transporter), member B3	0.7	0.0329
SLC35F5	Solute carrier family 35, member F5	1.0	0.0128
SLC37A1	Solute carrier family 37 (glucose-6-phosphate transporter), member 1	0.6	0.0245
SLC38A1	Solute carrier family 38, member 1	2.1	0.0027
SLC39A9	Solute carrier family 39, member 9	0.9	0.0053
ABCA11P	ATP binding cassette subfamily A member 11, pseudogene	2.3	0.0085
ABCE1	ATP binding cassette subfamily E member 1	1.0	0.026
TAP1	Transporter 1, ATP-binding cassette, sub-family B (MDR/TAP)	1.2	0.0024
ATP2B1	ATPase, Ca++ transporting, plasma membrane 1	1.2	0.0084
ATP5E	ATP synthase, H+ transporting, mitochondrial F1 complex, epsilon subunit	0.8	0.0011
ATP6AP1L	ATPase, H+ transporting, lysosomal accessory protein 1-like	0.7	0.011
ATP6V1B2	ATPase, H+ transporting, lysosomal 56/58kDa, V1 subunit B2	1.0	0.0011
ATP6V1E1	ATPase, H+ transporting, lysosomal 31kDa, V1 subunit E1	0.9	0.009
ATP8B1	ATPase, aminophospholipid transporter, class I, type 8B, member 1	1.7	0.0067
ATP10A	ATPase, class V, type 10A	1.8	0.0066
ATP11C	ATPase, class VI, type 11C	1.9	0.0003
ATP13A3	ATPase type 13A3	1.4	0.0003

CLIC4	Chloride intracellular channel 4	1.2	0.0074
NALCN	Sodium leak channel, non-selective	1.3	0.0009
SCN3A	Sodium channel, voltage gated, type III alpha subunit	1.1	0.0401
TRPC4	Transient receptor potential cation channel, subfamily C, member 4	2.3	9.20E-07
TRPC6	Transient receptor potential cation channel, subfamily C, member 6	0.7	0.0132
TRPM7	Transient receptor potential cation channel, subfamily M, member 7	1.2	0.0362
KCNJ6	Potassium channel, inwardly rectifying subfamily J, member 6	0.7	0.0261
KCNMB1	Potassium channel subfamily M regulatory beta subunit 1	1.5	0.0027
KCNT1	Potassium channel, sodium activated subfamily T, member 2	3.3	1.81E-09
TMC8	Transmembrane channel like 8	0.6	0.0439
ADORA2B	Adenosine A2b receptor	0.8	0.0336
CALCRL	Calcitonin receptor like receptor	1.4	0.0417
FZD6	Frizzled class receptor 6	1.4	2.48E-05
GPRC5A	G protein-coupled receptor, class C, group 5, member A	1.1	0.0042
GPR155	G protein-coupled receptor 155	1.3	0.0173
GPR180	G protein-coupled receptor 180	1.6	0.0002
LGR5	Leucine-rich repeat containing G protein-coupled receptor 5	1.0	0.0177
OPN3	Opsin 3	1.3	0.0068
PTGFR	Prostaglandin F receptor (FP)	2.8	9.21E-07

Comparison of gene expression data of co-culture vs. mono-culture of PCs in triplicates was performed by using Transcriptome Analysis Console (TAC, Applied Biosystems). For the analysis a fold change (FC) cut-off of 1.5 (≈log2FC +/- 0.59) and FDR p-value of 0.05 was applied.Log2 fold changes (log2 FC) and adjusted P-values (FDR p-value) are depicted in the third and fourth column, respectively.

Table S8: List of downregulated transporters, carriers, ion pumps and G protein-coupled receptors in pericytes co-cultured with endothelial cells vs. pericytes cultured alone.

**Gene**	**Gene description**	**log2 FC (co- vs. mono-culture)**	**FDR p-value**
SLC2A1	Solute carrier family 2 (facilitated glucose transporter), member 1/GLUT1	-0.7	0.0238
SLC6A6	Solute carrier family 6 (neurotransmitter transporter), member 6	-3.5	1.28E-08

SLC6A9	Solute carrier family 6 (neurotransmitter transporter, glycine), member 9/GLYT1	-1.2	0.0028
SLC9A3	Solute carrier family 9, subfamily A (NHE3, cation proton antiporter 3), member 3	-0.8	0.0181
SLC9A3R2	Solute carrier family 9, subfamily A (NHE3, cation proton antiporter 3), member 3 regulator 2	-1.1	0.0015
SLC9A9	Solute carrier family 9, subfamily A (NHE9, cation proton antiporter 9), member 9	-1.9	2.19E-06
SLC14A1	Solute carrier family 14 (urea transporter), member 1 (Kidd blood group)	-3.3	8.03E-08
SLC16A6	Solute carrier family 16, member 6	-1.5	0.0024
SLC20A1	Solute carrier family 20 (phosphate transporter), member 1	-2.3	5.72E-07
SLC25A6	Solute carrier family 25 (mitochondrial carrier; adenine nucleotide translocator), member 6	-2.0	2.85E-06
SLC25A38	Solute carrier family 25, member 38	-1.4	0.0094
SLC35E2B	Solute carrier family 35, member E2B	-1.7	6.96E-05
SLC35E2	Solute carrier family 35, member E2	-1.3	7.37E-05
SLC38A9	Solute carrier family 38, member 9	-1.3	0.0219
SLC44A1	Solute carrier family 44 (choline transporter), member 1	-0.9	0.0101
SLC44A4	Solute carrier family 44, member 4	-0.9	0.0024
SLCO5A1	Solute carrier organic anion transporter family, member 5A1	-0.8	0.0154
ABCF3	ATP binding cassette subfamily F member 3	-0.8	0.0482
ATP5D	ATP synthase, H+ transporting, mitochondrial F1 complex, delta subunit	-1.5	0.0005
ATP6V0A1	ATPase, H+ transporting, lysosomal V0 subunit a1	-1.0	0.0048
ATP6V0D1	ATPase, H+ transporting, lysosomal 38kDa, V0 subunit d1	-0.7	0.0447
ATP8B2	ATPase, aminophospholipid transporter, class I, type 8B, member 2	-0.7	0.0082
ATP9A	ATPase, class II, type 9A	-0.8	0.0047
ATP13A1	ATPase type 13A1	-0.8	0.0081
TPCN1	Two pore calcium channel protein 1	-0.8	0.0014
KCNMA1	Potassium channel, calcium activated large conductance subfamily M alpha, member 1	-1.8	0.0004
PIEZO2	Piezo-type mechanosensitive ion channel component 2	-1.8	8.31E-05

CMKLR1	Chemerin chemokine-like receptor 1	-1.1	0.0008
GPER	G protein-coupled estrogen receptor 1	-1.2	5.74E-05
GPR137B	G protein-coupled receptor 137B	-1.4	0.0017
S1PR3	Sphingosine-1-phosphate receptor 3	-0.9	0.0464

Comparison of gene expression data of co-culture vs. mono-culture of PCs in triplicates was performed by using Transcriptome Analysis Console (TAC, Applied Biosystems). For the analysis a fold change (FC) cut-off of 1.5 (≈log2FC +/- 0.59) and FDR p-value of 0.05 was applied. Log2 fold changes (log2 FC) and adjusted P-values (FDR p-value) are depicted in the third and fourth column, respectively.

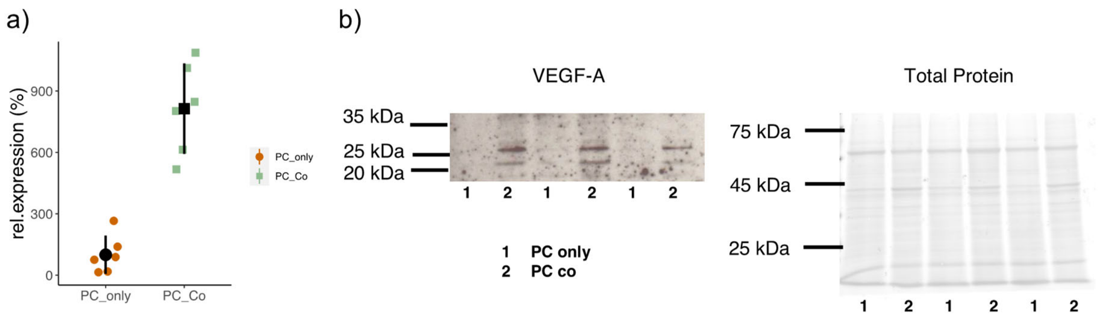

Figure S2: VEGF-A expression in mono- and co-culture pericytes (PCs).
PCs were cultured on Transwell inserts alone (PC only) or in co-culture with endothelial cells on the abluminal surface (PC co) for 7 days before they were trypsinized and lysed for Western blotting. VEGF-A expression levels are shown after normalization to total protein stain **(a)**. Immunoblots for VEGF-A (Santa Cruz, #sc-152, predicted molecular weight: 21-25 kDa) and for total protein stain (REVERT, total protein stain, LI-COR, #926-11011) are shown **(b)**. Experiment has been performed two times in triplicates and data represent mean +/- sd.

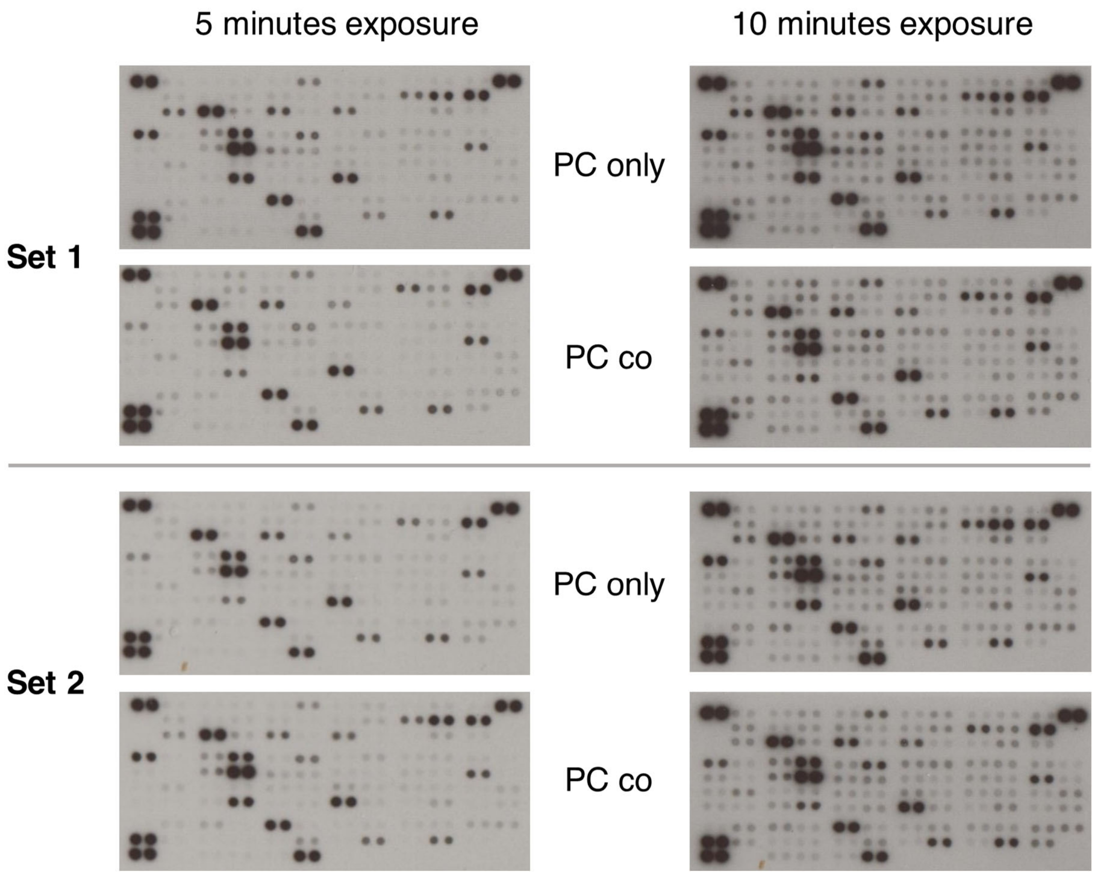

Figure S3: Array blots from Proteome Profiler Human XL Cytokine Array Kit.

Analysis was performed on cell lysates obtained from pericytes cultured alone (PC only) and in co-culture with endothelial cells (PC co). Experiments were performed two times with independent samples at two different exposure times of 5- and 10 min, in order to optimally determine expression levels of proteins with a high and low expression profile.

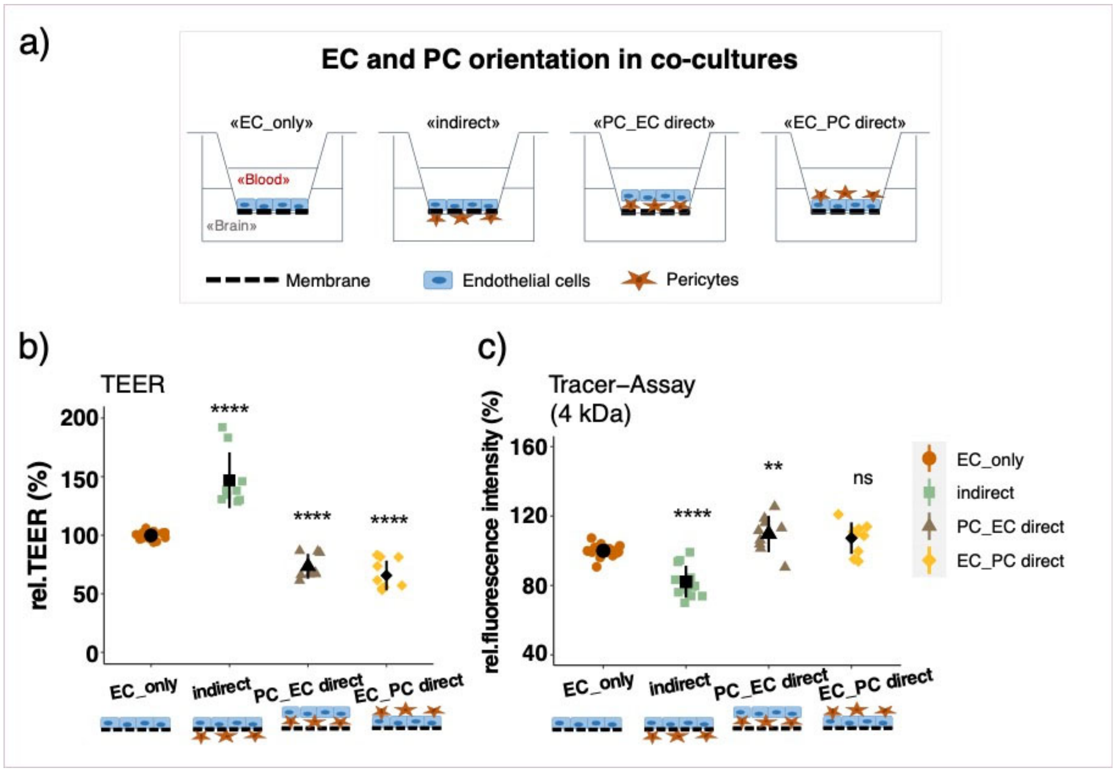

Figure S4: Measurements of endothelial barrier function with and without pericytes (PCs) in different constellations (recently published in *Cells* [18]). Endothelial cells (ECs) were cultured on Transwell inserts alone (EC_only), with PCs on the opposite side of the insert (indirect) or on the same side (PC_EC direct: PCs seeded first; and EC_PC direct: ECs seeded first) **(a)**. Cells were cultured for 7 days before barrier function was assessed. Relative trans-endothelial electric resistance (TEER) measurements with a CellZcope instrument **(b)**. Relative fluorescence intensity measured by macromolecular tracer assay with FITC-dextran 4 kDa **(c)**. Experiments were performed at least three times in triplicates and data represent mean +/- sd. ** $P < 0.01$, **** $P < 0.0001$, compared to "EC_only". Kruskal-Wallis rank sum test and subsequent pairwise Wilcoxon test with Benjamini–Hochberg corrections for multiple comparisons.

Supplementary Table S9: Differentially regulated genes (DRGs) that are common in endothelial cells (ECs) and pericytes (PCs) upon co-culture.

Gene	Gene description	Regulation in PCs	Regulation in ECs
AK5	Adenylate kinase 5	up	up
EFEMP1	EGF containing fibulin-like extracellular matrix protein 1	up	up
CFH	Complement factor H	up	up
TGFB2	Transforming growth factor beta 2	up	up
KCNT2	potassium channel, sodium activated subfamily T, member 2	up	up
EDIL3	EGF-like repeats and discoidin I-like domains 3	up	up
NEGR1	Neuronal growth regulator 1	up	up
TAGLN	Transgelin	up	up

ANKRD1	Ankyrin repeat domain 1 (cardiac muscle)	up	up
MGP	Matrix Gla protein	up	up
TCF4	Transcription factor 4	up	up
FOSL1	FOS-like antigen 1	down	down
MGST1	Microsomal glutathione S-transferase 1	down	down
EMP1	Epithelial membrane protein 1	down	down
RAC2	Ras-related C3 botulinum toxin substrate 2 (rho family, small GTP binding protein Rac2)	down	down
CYP1B1	Cytochrome P450, family 1, subfamily B, polypeptide 1	down	down
ANTXR2	Anthrax toxin receptor 2	down	down
LTBP1	Latent transforming growth factor beta binding protein 1	down	down
STC2	Stanniocalcin 2	down	down
NFE2L3	Nuclear factor, erythroid 2-like 3	down	down
G0S2	G0/G1 switch 2	down	down
ISG15	ISG15 ubiquitin-like modifier	up	down
IFI44	Interferon-induced protein 44-like	up	down
FABP3	Fatty acid binding protein 3, muscle and heart	up	down
SCP2	Sterol carrier protein 2	up	down
MTHFD2	Methylenetetrahydrofolate dehydrogenase (NADP+ dependent) 2, methenyltetrahydrofolate cyclohydrolase	up	down
CMPK2	Cytidine monophosphate (UMP-CMP) kinase 2, mitochondrial	up	down
SCG2	Secretogranin II	up	down
DOCK10	Dedicator of cytokinesis 10	up	down
RSAD2	Radical S-adenosyl methionine domain containing 2	up	down
PTX3	Pentraxin 3, long	up	down
MINA	MYC induced nuclear antigen	up	down
NCEH1	Neutral cholesterol ester hydrolase 1	up	down
CLDN1	Claudin 1	up	down
CXCL8	Chemokine (C-X-C motif) ligand 8	up	down
CXCL5	Chemokine (C-X-C motif) ligand 5	up	down
CXCL10	Chemokine (C-X-C motif) ligand 10	up	down
CXCL6	Chemokine (C-X-C motif) ligand 6	up	down
GPX8	Glutathione peroxidase 8 (putative)	up	down
POLR3G	Polymerase (RNA) III (DNA directed) polypeptide G (32kD)	up	down
BAG2	BCL2-associated athanogene 2	up	down
TUBE1	Tubulin, epsilon 1	up	down
EEF1E1	Eukaryotic translation elongation factor 1 epsilon 1	up	down
HDAC9	Histone deacetylase 9	up	down
INSIG1	Insulin induced gene 1	up	down
PARP12	Poly(ADP-ribose) polymerase family member 12	up	down
DDX58	DEAD (Asp-Glu-Ala-Asp) box polypeptide 58	up	down
PLAU	Plasminogen activator, urokinase	up	down

IFIT2	Interferon-induced protein with tetratricopeptide repeats 2	up	down
IFIT3	Interferon-induced protein with tetratricopeptide repeats 3	up	down
IFIT1	Interferon-induced protein with tetratricopeptide repeats 1	up	down
IDI1	Isopentenyl-diphosphate delta isomerase 1	up	down
GLIPR1	GLI pathogenesis-related 1	up	down
SOCS2	Suppressor of cytokine signaling 2	up	down
RRAS2	Related RAS viral (r-ras) oncogene homolog 2	up	down
OAS1	2-5-oligoadenylate synthetase 1	up	down
IFI27	Interferon, alpha-inducible protein 27	up	down
TRIM69	Tripartite motif containing 69	up	down
ADORA2B	Adenosine A2b receptor	up	down
USP18	Ubiquitin specific peptidase 18	up	down
BID	BH3 interacting domain death agonist	up	down
USP41	Ubiquitin specific peptidase 41	up	down
ABLIM1	Actin binding LIM protein 1	down	up
METTL7A	Methyltransferase like 7A	down	up
EFNA5	Ephrin-A5	down	up
LPCAT2	Lysophosphatidylcholine acyltransferase 2	down	up

In total, 66 DRGs were common in the two cell types, as is visible from the Venn Diagram in Figure 6. The regulation of genes in co-cultured vs. mono-cultured cells is denoted in the third and fourth columns, respectively. Comparison of gene expression data of co-cultured vs. mono-cultured PCs and ECs, respectively, in triplicates was performed by using Transcriptome Analysis Console (TAC, Applied Biosystems). For the analysis a fold change (FC) cut-off of 1.5 (≈log2FC +/- 0.59) and FDR p-value of 0.05 was applied.

Supplementary Table S10: Pathway enrichment analysis (BioPlanet)of differentially regulated genes (DRGs) that are common in ECs and PCs.

**Pathway**	**Overlap**	**adj. P-value**
Interferon alpha/beta signaling	7/64	7.21E-11
Interferon signaling	8/168	2.37E-09
Immune system signaling by interferons, interleukins, prolactin, and growth hormones	8/280	1.27E-07
Interleukin-1 regulation of extracellular matrix	6/120	2.09E-07
FRA pathway	4/37	1.16E-06
TGF-beta regulation of extracellular matrix	9/565	2.57E-06
Type II interferon signaling (interferon-gamma)	4/50	3.97E-06
Binding of chemokines to chemokine receptors	4/54	5.42E-06
Antiviral mechanism by interferon-stimulated genes	4/70	1.53E-05
RIG-I-like receptor signaling pathway	4/71	1.62E-05

Analysis was performed using NCATS BioPlanet on the Enrichr website with DRGs. Second column describes number of commonly regulated genes compared to total number of genes in the pathway. P-values adjusted for multiple testing (adj. P-value) are noted in the last column.

Supplementary Table S11: Differential regulation of interferon-induced genes in co-cultured PCs and ECs.

Gene	Gene description	Regulation in PCs	Regulation in ECs
IFIT1	Interferon-induced protein with tetratricopeptide repeats 1	+8.5	-1.7
IFI44	Interferon-induced protein 44-like	+2.5	-1.1
IFI27	Interferon, alpha-inducible protein 27	+4.0	-1.1
IFIT2	Interferon-induced protein with tetratricopeptide repeats 2	+2.0	-1.9
IFIT3	Interferon-induced protein with tetratricopeptide repeats 3	+3.5	-1.7

Interferon-induced genes that are regulated in both, PCs and ECs upon co-culture. Comparison of gene expression data of co-cultured vs. mono-cultured PCs and ECs, respectively, in triplicates was performed by using Transcriptome Analysis Console (TAC, Applied Biosystems). For the analysis a fold change (FC) cut-off of 1.5 (≈log2FC +/- 0.59) and FDR p-value of 0.05 was applied.
